# Determining incremental coulombic efficiency and physiological parameters of early stage *Geobacter* spp. enrichment biofilms

**DOI:** 10.1371/journal.pone.0234077

**Published:** 2020-06-19

**Authors:** Benjamin Korth, Jörg Kretzschmar, Manuel Bartz, Anne Kuchenbuch, Falk Harnisch

**Affiliations:** 1 Department of Environmental Microbiology, Helmholtz Centre for Environmental Research—UFZ, Leipzig, Saxony, Germany; 2 Biochemical Conversion Department, DBFZ Deutsches Biomasseforschungszentrum gemeinnützige GmbH, Leipzig, Saxony, Germany; University of Notre Dame, UNITED STATES

## Abstract

*Geobacter* spp. enrichment biofilms were cultivated in batch using one-chamber and two-chamber bioelectrochemical reactors. Time-resolved substrate quantification was performed to derive physiological parameters as well as incremental coulombic efficiency (i.e., coulombic efficiency during one batch cycle, here every 6h) during early stage biofilm development. The results of one-chamber reactors revealed an intermediate acetate increase putatively due to the presence of acetogens. Total coulombic efficiencies of two-chamber reactors were considerable lower (19.6±8.3% and 49.3±13.2% for 1^st^ and 2^nd^ batch cycle, respectively) compared to usually reported values of mature *Geobacter* spp. enrichment biofilms presumably reflecting energetic requirements for biomass production (i.e., cells and extracellular polymeric substances) during early stages of biofilm development. The incremental coulombic efficiency exhibits considerable changes during batch cycles indicating shifts between phases of maximizing metabolic rates and maximizing biomass yield. Analysis based on Michaelis-Menten kinetics yielded maximum substrate uptake rates (*v*_max,Ac_, *v*_max,I_) and half-saturation concentration coefficients (*K*_M,Ac_,*K*_M,I_) based on acetate uptake or current production, respectively. The latter is usually reported in literature but neglects energy demands for biofilm growth and maintenance as well as acetate and electron storage. From 1^st^ to 2^nd^ batch cycle, *v*_max,Ac_ and *K*_M,Ac_, decreased from 0.0042–0.0051 mmol Ac^−^ h^−1^ cm^−2^ to 0.0031–0.0037 mmol Ac^−^ h^−1^ cm^−2^ and 1.02–2.61 mM Ac^−^ to 0.28–0.42 mM Ac^−^, respectively. Furthermore, differences between *K*_M,Ac_/*K*_M,I_ and *v*_max,Ac_/*v*_max,I_ were observed providing insights into the physiology of *Geobacter* spp. enrichment biofilms. Notably, *K*_M,I_ considerably scattered while *v*_max,Ac_/*v*_max,I_ and *K*_M,Ac_ remained rather stable indicating that acetate transport within biofilm only marginally affects reaction rates. The observed data variation mandates the requirement of a more detailed analysis with an improved experimental system, e.g., using flow conditions and a comparison with *Geobacter* spp. pure cultures.

## Introduction

Electroactive microorganisms (EAM) possess the metabolic trait of performing extracellular electron transfer (EET) [1]. EET can be defined as the capability of either donating electrons to insoluble reactants serving as terminal electron acceptors (TEA, e.g., metal ores and electrodes) or receiving electrons therefrom. Several mechanisms of EET are known [2,3] that can be assigned to either (i) direct extracellular electron transfer (DEET) or to (ii) mediated extracellular electron transfer (MEET). DEET requires a direct physical contact between microorganisms and insoluble TEA. The contact is facilitated by outer membrane-bound cytochromes and/ or by conductive biological structures termed nanowires transferring electrons across several dozens of micrometers (long-range DEET). MEET is based on electron transport via soluble redox shuttles like flavins [4] or phenazines [5]. Further, electrons can be transferred between phylogenetically different microorganisms with one being the electron donor and the other the electron acceptor [6]. This is termed direct interspecies electron transfer (DIET) and enables the creation of metabolic webs. Another related type of electron conduction was discovered in *Desulfobulbaceae*. Here, by forming multicellular filaments, electrons are transported across distances of few centimeters connecting, for instance, oxic and anoxic zones of marsh [7].

In addition to their currently discussed ecological relevance, e.g., for biogeochemical redox cycles [8,9] and the degradation of organic matter [10], EAM are also proposed and intensively studied as bioelectrocatalysts in microbial electrochemical technologies (MET) for, e.g., wastewater treatment, current production, biological sensors, desalination, and chemical synthesis [1,11–13].

EAM are no (phylogenetically) distinct group of microorganisms and do occur in different ecological niches [14]. Some species of the genus *Geobacter* are of special interest as they utilize a certain variety of electron donors and TEA [15–18]. Moreover, few members of this genus form multilayered biofilms at anodes and produce current densities of up to 0.8–1.0 mA cm^−2^ [19,20]. Therefore, *Geobacter* species are used as model organisms for studying DEET in terms of electron transfer kinetics [21], biochemical properties of redox centers [22,23], thermodynamics (including analyses of Gibbs free energy and enthalpy) [24,25], and biofilm conductivity [26,27].

Typical electrochemical parameters for assessing the performance of EAM at electrodes are maximum current density (*j*_max_, maximum current normalized to projected surface area of the electrode) and coulombic efficiency (CE). CE is a measure of the ratio of theoretically and actually transferred number of electrons at the electrode. Hence it can be regarded as measure for the overall electron efficiency of EAM [28]. In case of biofilm anodes, it describes the fraction of electrons that is derived from substrate oxidation and finally transferred to the anode (Eq 1). Consequently, CE also provides quantitative information about the role of undesired side reactions, i.e., utilization of soluble TEA, biomass formation as well as substrate competition, e.g., by acetoclastic methanogenesis.

$$CE=\frac{\int Idt}{{\Delta n}_{S}zF}$$ (1)

*I* is current, *t* is time, Δ*n*_S_ is amount of consumed substrate, *z* is number of electrons in the substrate, *F* is the Faraday constant (96485.3 C mol^−1^)–see Supporting Information for a list of symbols and constants.

**Fig 1:**
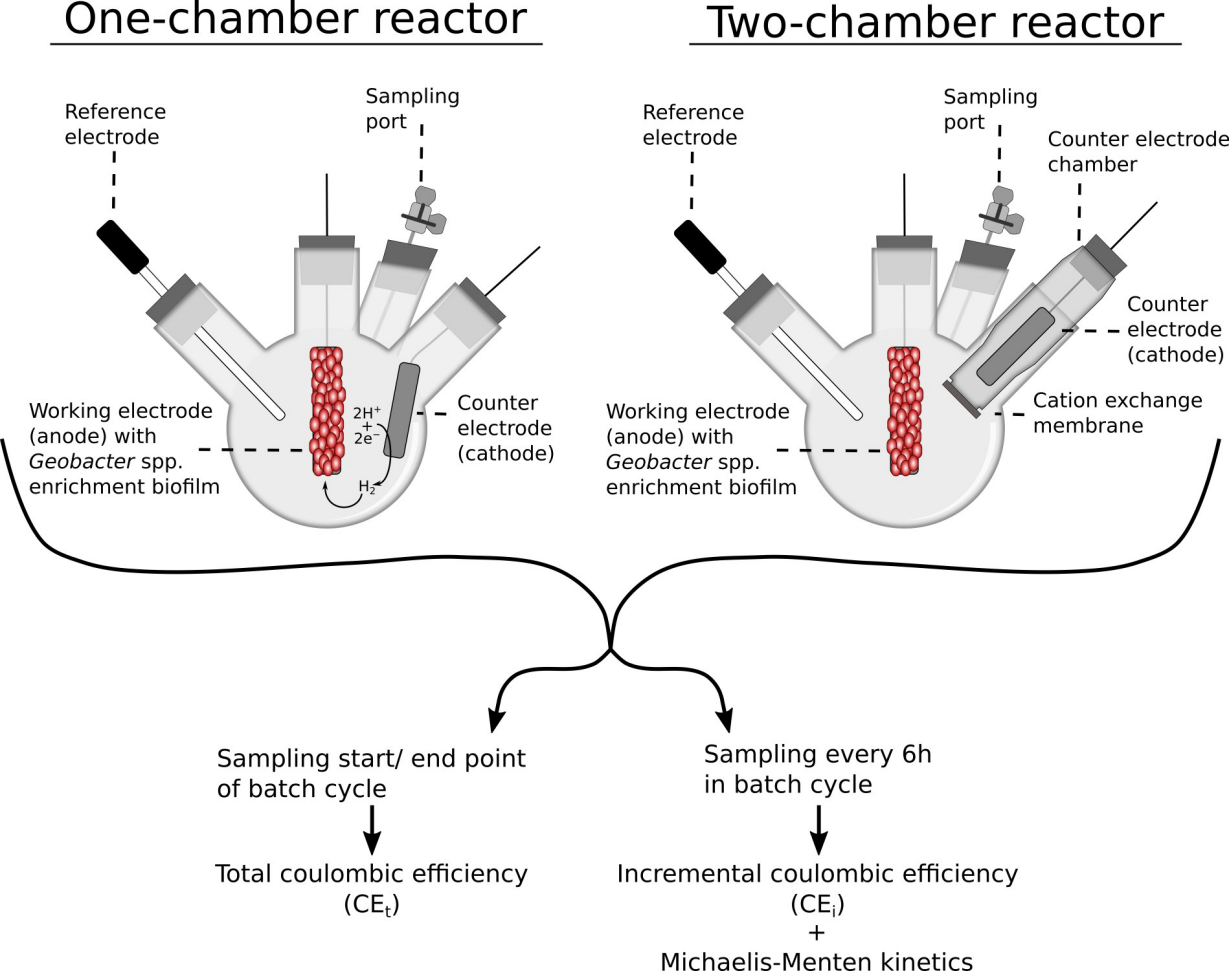
Schematic illustration of the experimental setup. A three-electrode setup and sampling port were incorporated in four-neck round-bottom flasks. In one-chamber reactors, working and counter electrode are located in one compartment. Therefore, *Geobacter* spp. enrichment biofilms can consume cathodically produced hydrogen. In two-chamber reactors, a counter electrode chamber with a fixed cation exchange membrane is introduced minimizing crossover of cathodically produced hydrogen. All reactors were sampled at the beginning as well as at the end of a batch cycle and every 6 h during a batch cycle for obtaining total (CE_t_) and incremental coulombic efficiencies (CE_i_), respectively.

According to literature, CE of mature *Geobacter sulfurreducens* and *Geobacte*r spp. enrichment biofilms are both in the range of 80–95% [29–32]. Yet, to determine CE, the configuration of the electrochemical cell forming the bioelectrochemical reactor has to be considered. For fundamental as well as engineering studies, often bioelectrochemical reactors with three-electrode configuration and potentiostatic control are used. Thereby, bioelectrochemical reactors without separation of anode and cathode (i.e., one-chamber reactors) as well as with separation of anode and cathode compartment with an ion exchange membrane (i.e., two-chamber reactors) are used. As depicted in Fig 1, when cultivated at anodes in one-chamber reactors, cathodically produced hydrogen can be utilized by *Geobacter* spp. [15,33]. This “feeding” by the cathode is considerably reduced in two-chamber reactors. In the majority of cases, *Geobacter* spp. are cultivated at anodes in batch mode. Thereby, the CE is calculated for complete batch cycles, i.e., by sampling the start and end-point (Table 1, see also Fig 1). This total CE (CE_t_) provides information about the efficiency of current production of the entire batch cycle. However, CE_t_ does not reflect changes in CE during the batch cycle, e.g., due to biofilm development and changing cultivation conditions (e.g., acetate (Ac^−^) concentration). In contrast, a time-resolved CE analysis during batch cycles, i.e., incremental CE (CE_i_), might provide further insights into the physiology. For instance, CE_i_ could indicate the existence of a metabolic shift of *Geobacter* spp. focusing either on current production (i.e., optimizing metabolic rates) or on formation of cells and extracellular polymeric substances (i.e., optimizing biomass yield) during a batch cycle. This could indicate selection between two distinct microbial lifestyles [34], however, the data foundation on CE_i_ is rather scarce. Geelhoed et al. described an increase of CE_i_ from 16 to 66% during anodic polarization of *Geobacter sulfurreducens* PCA based on 7 data points within 3 days [35]. Lee et al. showed for a few values that the CE of a *Geobacter* dominated electroactive biofilm in a one-chamber flow cell is depending on the hydraulic retention time [33].

**Table 1:** List of total coulombic efficiencies derived from studies cultivating *Geobacter sulfurreducens* and *Geobacter* spp. enrichment biofilms in one- and two-chamber reactors.

Microorganism	Experimental system^a^	Duration of cultivation [d]	Total coulombic efficiency [%]	Reference
*Geobacter sulfurreducens* PCA	Two-chamber reactor, MEC, *E*_A_ = 0.40 V	6.5	97	[30]
*Geobacter sulfurreducens* PCA	Two-chamber reactor, MFC	>60	100	[36]
*Geobacter sulfurreducens* PCA	Two-chamber reactor, mixed MFC/MEC,	5	34^b^	[35]
*Geobacter sulfurreducens* PCA	Two-chamber reactor, MEC, *E*_A_ = 0.45 V	4	80	[29]
*Geobacter* spp. enrichment biofilm	Two-chamber reactor, MFC	>30	72	[37]
*Geobacter* spp. enrichment biofilm	Two-chamber reactor, MEC, *E*_A_ = −0.16 V	28	98	[38]
*Geobacter sulfurreducens* PCA	One-chamber reactor, MEC, *E*_A_ = 0.60 V	>10	89	[39]
*Geobacter metallireducens* GS-15	One-chamber reactor, MEC, *E*_A_ = 0.60 V	>10	82	[39]
*Geobacter* spp. enrichment biofilm	One-chamber reactor, MEC, *E*_A_ = 0.40 V	2–160	89^b^	[32]
*Geobacter* spp. enrichment biofilm	One-chamber reactor, MEC, *E*_A_ = 0 V	NM	60–140	[40]

^a^All provided potentials refer to the standard hydrogen electrode, MEC: microbial electrolysis cell, MFC: microbial fuel cell, *E*_A_: anode potential

^b^Average CE during anode polarization

NM not mentioned

In addition to the above mentioned knowledge, determining CE_i_ with a time-resolved measurement based on substrate uptake, i.e., acetate consumption, provides the further opportunity to determine physiological parameters of *Geobacter* spp. biofilms that were only scarcely available, so far. A well-accepted physiological characterization is using Michaelis-Menten kinetics (Eq 2). It was initially developed for enzymatic reactions but this approach is also suitable for empirically investigating microbial cultures [41] and was already applied to EAM for a few times (Table 2). By relating substrate uptake rate with substrate concentration, maximum substrate uptake rate (*v*_max_) and the concentration at which substrate uptake rate is $\frac{v_{max}}{2}$ (half-saturation concentration, *K*_M_) can be derived.

$$v=v_{max}\frac{C_{S}}{C_{S}+K_{M}}$$ (2)

*v* is substrate uptake rate, *C*_S_ is substrate concentration

**Table 2:** List of studies that applied Michaelis-Menten kinetics to Geobacter sulfurreducens and Geobacter spp. enrichment biofilms and determined *K*_*M*_ and *v*_*max*_ values.

Microorganism	Experimental system	*K*_M_	*v*_max_	Reference
*Geobacter sulfurreducens*	Planktonic culture, fumarate as TEA, batch mode	0.03 mM Ac^−^	4.5 mmol Ac^−^ g_DW_^−1^ h^−1^	[42]
*Geobacter sulfurreducens*	Planktonic culture, Fe(III)-citrate as TEA, batch mode	0.01 mM Ac^−^	14.8 mmol Ac^−^ g_DW_^−1^ h^−1^	[42]
*Geobacter sulfurreducens* PCA	Biofilm anode in one-chamber reactor, flow mode, > 12 days of cultivation	0.59 mM Ac^−^	ND	[43]^a^
*Geobacter sulfurreducens* PCA	Biofilm anode in one-chamber reactor, batch mode, > 12 days of cultivation	0.62 mM Ac^−^	0.0114 mmol Ac^−^ cm^−2^ h^−1^	[43]^a^^,^^b^
*Geobacteraceae* enrichment biofilm	Biofilm anode in one-chamber reactor, flow mode, 60 days of cultivation	1.86–2.86 mM Ac^−^	12.88–15.41 mmol Ac^−^ cm^−2^ h^−1^	[44]^a^^,^^b^
*Geobacter* spp. enrichment biofilm	Biofilm anode in one-chamber reactor, batch mode, 1 month of cultivation	0.67 mM Ac^−^	0.00043 mmol Ac^−^ cm^−2^ h^−1^	[45]^a^^,^^b^
*Geobacter* spp. enrichment biofilm	Biofilm anode in one-chamber reactor, flow mode, > 14 days of cultivation	1.43 mM Ac^−^	0.0019 mmol Ac^−^ cm^−2^ h^−1^	[13]^a^^,^^b^

^a^Data is based on current production and not on acetate (Ac^−^) uptake

^b^Values were converted from results of respective publication (see S1 Literature data and S2–S4 Figs in S1 File for details)

ND not determined

It is of note that the parameters listed in Table 2 are all based on current production and not on acetate uptake (i.e., *v*_max,I_, see Material & Methods). Thus, an immediate link between acetate uptake and EET (with CE = 100%) is assumed. Therefore, microbial processes like biomass formation, biomass maintenance as well as acetate [46] and electron storage [47] are not considered in these analyses, and thus the derived physiological parameters could be biased. An improved physiological characterization of *Geobacter* spp. enrichment biofilms by simultaneously analyzing acetate uptake, *j*, CE_t_, CE_i_, *v*_max_, and *K*_M_ would provide a better understanding of substrate and/or electron storage processes as well as putative metabolic shifts of *Geobacter* spp. during biofilm growth. For instance, by comprehensively characterizing substrate uptake of electroactive biofilms, the start-up phase of MET could be specifically designed avoiding the current erratic behavior thereof [48–50]. Therefore, a study combining time-resolved substrate analysis during batch cultivation with electrochemical measurements of *Geobacter* spp. enrichment biofilms was performed. The experiments were conducted in one-chamber and two-chamber reactors. Experiments in one-chamber reactors were biased by cathodically produced hydrogen [40] but allowed an evaluation of the influence thereof on CE_i_, *v*_max_, and *K*_M_ of acetate-fed *Geobacter* spp. enrichment biofilms. The analysis was restricted to the first two batch cycles for focusing on the physiological development of early stage *Geobacter* spp. enrichment biofilms.

## Material & methods

### General conditions

All chemicals were of analytical or biochemical grade. If not stated otherwise, all provided potentials refer to the standard hydrogen electrode (SHE) by conversion from Ag/AgCl sat. KCl reference electrodes (+0.197 V vs. SHE).

### Reactor setup

The reactors were based on a three-electrode setup incorporated in four-neck round-bottom flasks (Lenz Laborglas GmbH & CO.KG, Germany) with 29/32 ISO 383 (ISO K-6 series) ground glass joints. In case of two-chamber reactors, a tailor-made glass tube with 29/32 ISO 383 ground glass joint and a junction for 20 mm aluminium crimp cap (WICOM Germany GmbH, Germany) was inserted representing the counter electrode chamber (Fig 1). A cation exchange membrane (*A* = 1 cm^2^, fumasep®FKE, FuMA-Tech GmbH, Germany) was fixed with a butyl rubber O-ring and an aluminium crimp cap to the 20 mm junction. The ground glass joint of the glass tube was sealed with PTFE ground joint sleeve (Lenz Laborglas GmbH & CO.KG, Germany). The remaining ground glass joints were closed with silicone stoppers (Deutsch & Neumann GmbH, Germany). The working electrode (WE) consisted of a stainless steel wired (*d* = 0.6 mm, Goodfellow GmbH, Germany) graphite rod (*d* = 10 mm, *L* = 40 mm, *A* = 13.4 cm^2^, quality CP-2200, CP-Graphitprodukte GmbH, Germany). The connection was sealed with epoxy resin (HT2, R&G Faserverbundwerkstoffe, Germany) and the wire was insulated with a polyolefin shrink tube (ABB Ltd., Switzerland). The wire was pulled through the silicone stopper. The counter electrode, i.e., cathode, was prepared identically (*d* = 10 mm, *L* = 50 mm, *A* = 16.5 cm^2^). The reference electrode (Ag/AgCl sat. KCl, +0.197 V vs. SHE, SE 11, Xylem Analytics Germany Sales GmbH & Co. KG Sensortechnik Meinsberg, Germany) was passed gastight through a tailored silicone stopper. The electrode assembly and the distances between electrodes were identical for all reactors (4 cm). The remaining fourth stopper was pierced with a sterile cannula (Sterican®, B. Braun Melsungen AG, Germany) connected to three-way valve (Discofix®, B. Braun Melsungen AG) for liquid sampling (Fig 1). A magnetic stirrer was operated with 150 rpm. Reactors, stirrers, and stoppers were autoclaved prior use. The prepared cathode chamber and all electrodes were treated with sterilizing solution (675 mL of 96% ethanol mixed with 6.75 mL of concentrated sulfuric acid and filled to 1 L with ultrapure water) for 30 min and then washed with sterile ultrapure water.

### Biofilm cultivation

Experiments were conducted in repeated batch mode. Prepared reactors were filled with 250 mL medium according to Kim et al. [51]. Briefly, the medium was prepared with 0.82 g L^−1^ sodium acetate, 2.69 g L^−1^ NaH_2_PO_4_×H_2_O, 4.33 g L^−1^ Na_2_HPO_4_, 0.3 g L^−1^ NH_4_Cl, 0.13 g L^−1^ KCl, 12.5 mL L^−1^ vitamin solution, and 12.5 mL L^−1^ trace metal solution [52]. Medium was purged via sterile cannula and sterile filter (Labsolute®, Th. Geyer GmbH & Co. KG, Germany) with nitrogen (purity 99.999%) for 30 min for obtaining anaerobic conditions. For inoculation, a *Geobacter* spp. enrichment biofilm obtained as described previously was used [53]. In brief, a wastewater derived *Geobacter* spp. enrichment biofilm was cultivated for 4 batch cycles in a one-chamber reactor like described above and biofilm samples were collected with a sterile cannula and resuspended in 5 mL deaerated medium. The biofilm was homogenized by vortexing and flushing with nitrogen. For inoculation, 300 μL of this suspension was added to each reactor. Afterwards, medium was purged with nitrogen for additional 10 min via sterile cannula and sterile filter. The cathode chamber was filled with 15 mL medium but without sodium acetate. All reactors were cultivated at 35°C. Medium was exchanged when current dropped to zero due to substrate depletion. 250 mL fresh medium was sterilely purged with nitrogen for 30 min in a laboratory bottle (SCHOTT AG, Germany). Reactors and fresh medium were steadily purged with nitrogen during medium exchange. Medium was carefully decanted and after refilling with fresh medium, the reactors were purged for additional 10 min. WE were poised at +397 mV and current was recorded every 600 s using a multipotentiostat (MPG-2, Bio-Logic Science Instruments, France). Every 24 h cyclic voltammograms were acquired with a scan rate of 1 mV s^−1^ between −303 mV and +497 mV (each with 3 consecutive scans and the 3^rd^ scan was used for analysis).

### Sampling

1 mL liquid samples were taken from fresh and used medium, centrifuged at 6.000×g and 4°C for 10 min. The supernatant was used for pH (SevenExcellence pH/Cond meter S470, Mettler-Toledo International Inc., USA) and HPLC measurements. Every 6 h, 500 μL samples were taken with a 1 mL syringe (Omnifix®, B. Braun Melsungen AG) from reactors via three-way valve. 300 μL were discarded for removing dead liquid volume, 200 μL were filtered (0.2 μm, PTFE, VWR International GmbH, Germany) and used for HPLC analysis.

### Microbial community analysis

Biofilm samples were taken with a sterile cannula directly from the electrode (n = 2 for each reactor). Genomic DNA was extracted with the NuceloSpin Tissue Kit (Macherey-Nagel, Germany). The microbial community composition on DNA level was analysed with a standard TRFLP procedure using the primers UniBac27f (FAM labelled) and Univ1492r for amplifying the partial sequence of the 16S rRNA gene of bacteria [54].

The PCR Master Mix contained 6.25 μL enzyme mix (MyTaq HS Red Mix, 2x,Bioline, Germany), 0.25 μL of each primer (5 ρmol μL^−1^, MWG Biotech, Germany), 4.75 μL nuclease-free water, and 1 μL genomic DNA (about 360–720 ng). The PCR cycle parameters were as follows: 1 min at 95°C, 25 cycles of 15 s at 95°C, 15 s at 54°C, and 2 min at 72°C, followed by a 10 min final extension step at 72°C [55]. PCR products were purified (Sure Clean Plus, Bioline) and digested with restriction endonucleases HaeIII and RsaI (New England Biolabs GmbH, Germany). After product precipitation (EDTA/EtOH), TRFLP analysis was performed using an ABI PRISM Genetic Analyzer 3130xl (Applied Biosystems, USA) and MapMarker® 1000 (BioVentures Inc., USA) as size standard.

### Acetate quantification

The acetate concentration of the medium was measured using high-performance liquid chromatography (HPLC, Shimadzu Scientific Instruments, Japan) equipped with a refractive index detector RID-10A and a HiPlex H column (300 × 7.7 mm, 8 mm pore size, Agilent Technolgies, USA) with a pre-column SecurityGuard Cartidge Carbo-H (4 × 3.0 mm, Phenomenex, USA). The liquid phase was 5 mM sulphuric acid and samples were run at a flow rate of 0.5 mL min^−1^ and with a temperature of 50°C for 30 min. The acetate concentration was determined based on a three point external standard calibration of the peak area (*R*^2^ > 0.99).

### Data analysis

**Fig 2:**
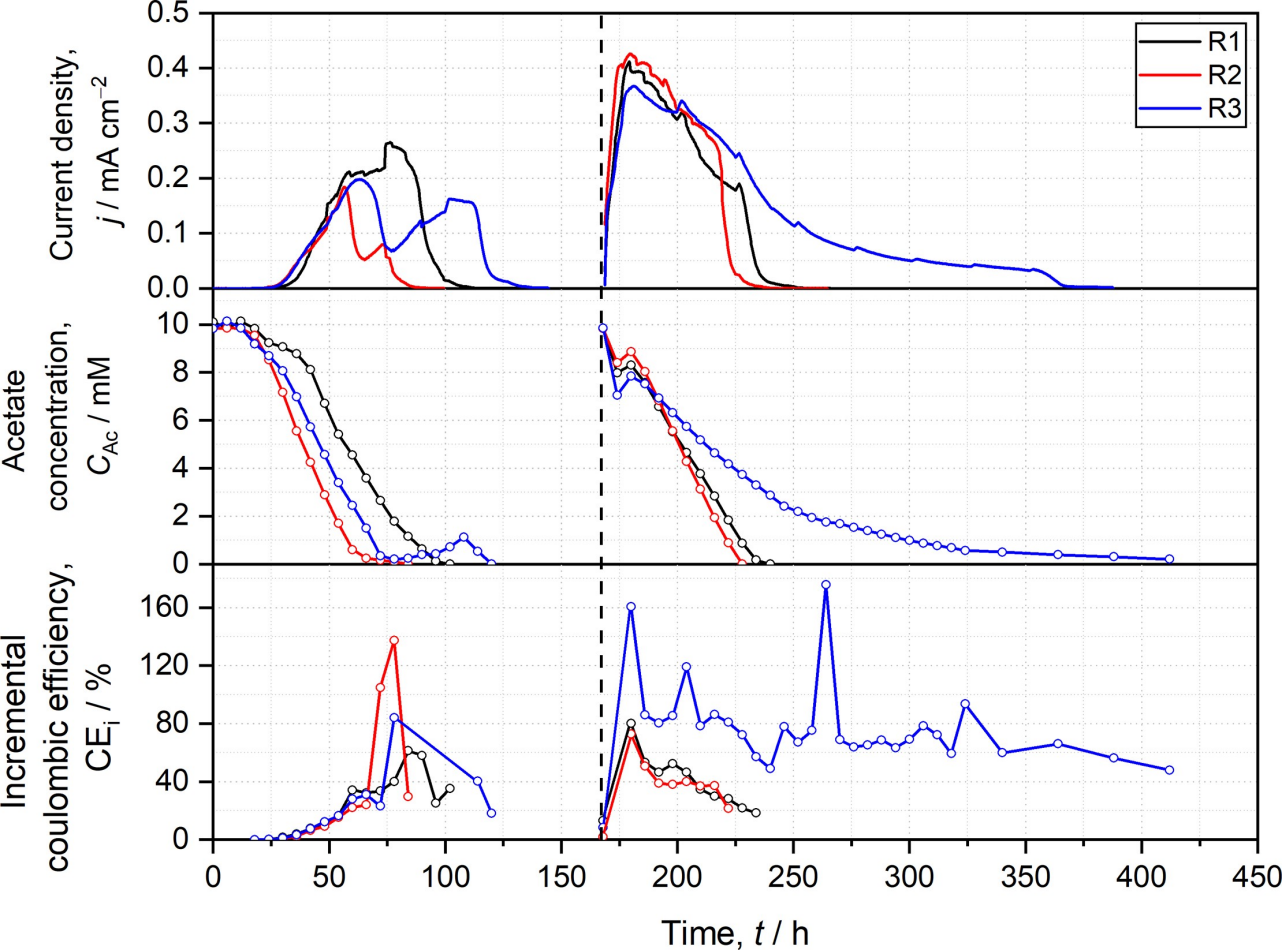
Temporal progressions of current density (*j*), acetate concentration (*C*_*Ac*_), and incremental coulombic efficiency (CEi) of two-chamber reactors. R1-3 represent experimental replicates. Open circles indicate sampling events. Vertical dashed line indicates beginning of 2^nd^ batch cycle after medium exchange.

**Fig 3:**
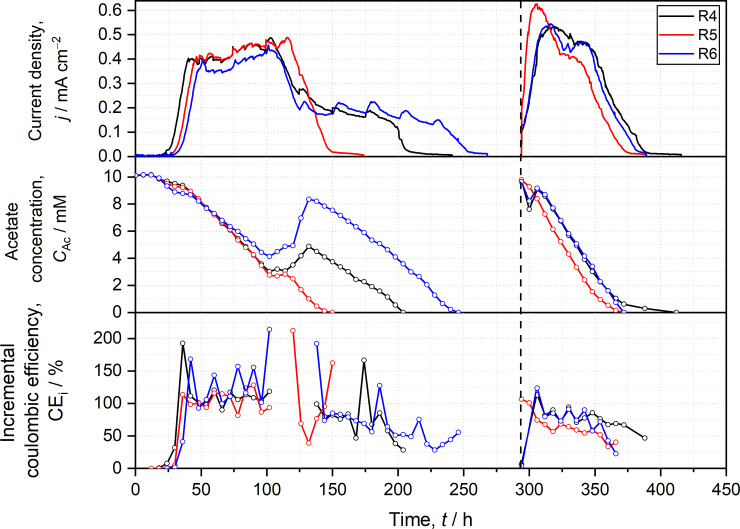
Temporal progressions of current density (*j*), acetate concentration (*C*_*Ac*_), and incremental coulombic efficiency (CEi) of one-chamber reactors. R4-6 represent experimental replicates. Open circles indicate sampling events. Vertical dashed line indicates beginning of 2^nd^ batch cycle after medium exchange.

The profiles of current density (*j*), acetate concentration (*C*_Ac_), and incremental coulombic efficiency (CE_i_) of two-chamber reactors (Fig 2) as well as one-chamber reactors (Fig 3) were manually synchronized (i.e., 2^nd^ batch cycles of all replicates start at the same time in the Figures although this was not the case during the experiment) for achieving a better visualization and comparability of data. Both CE were calculated from consumed acetate as well as produced charge within the respective time period (*t*, 6 h for CE_i_ and duration of the full batch cycles for CE_t_) according to Eq 3.

$$CE=\frac{\int_{0}^{t}Idt}{\Delta C_{Ac}\times V\times z\times F}$$ (3)

Δ*C*_Ac_ is change in acetate concentration, *V* is liquid volume, and *z* is number of electrons in acetate (8 electrons). *V* was always corrected for the removed volume (500 μL per sampling).

Acetate uptake rate (*v*_Ac_) was calculated with Eq 4.

$$v_{Ac}=\frac{\Delta C_{Ac}\times V}{A\times t}$$ (4)

*A* is anode area.

Current production was normalized to acetate equivalents (*v*_I_) via Eq 5. Thus, *v*_I_ represented an “acetate output rate”, assuming the absence of alternative electron donors, e.g., hydrogen.

$$v_{I}=\frac{\int_{o}^{t}Idt}{A\times z\times F\times t}$$ (5)

Subsequently, acetate concentration, *v*_Ac_, and *v*_I_ data were fitted with Michaelis-Menten equation for deriving *v*_max,Ac_, *v*_max,I_, *K*_M,Ac_, and *K*_M,I_ (Eq 2, see also S13 and S14 Figs in S1 File) being based on either acetate uptake or current. Michaelis-Menten kinetics was only applied to data points being subject to maximal biofilm activity, i.e., maximal acetate uptake rate was reached (S5 and S6 Figs in S1 File). Please note that the determined *K*_M,i_ and *v*_max,i_ values represent apparent Michaelis-Menten constants being subject to substrate depletion, biofilm thickness, biofilm density, surface area of electrodes, and convection.

## Results & discussions

### Microbial community analysis and electrochemical biofilm characterization

Electrochemical enrichment of *Geobacter* spp. biofilms at anodes, as done for this study, is widespread in microbial electrochemistry [38,39,44,45]. To assure that the further studied biofilm anodes are representative, fundamental electrochemical characterization as well as microbial analysis are performed. TRFLP analysis of the biofilms reveals that the *Geobacter* spp. amplicons have the highest relative abundance in biofilm anodes of two-chamber (87.3 ±13.3%, n = 4) and one-chamber (85.9 ±12.8%, n = 6) reactors (S9A Fig in S1 File). Furthermore, only one major formal potential (i.e., redox potential of active redox centers of biofilm anodes performing EET) is identified by applying cyclic voltammetry. The values of −115 ±23 mV (n = 19) for two-chamber reactors and −116 ±14 mV (n = 27) for one-chamber reactors clearly show that *Geobacter* spp. represent the dominating EAM (S10A Fig in S1 File) [56]. Thus, it is evident that the data derived in the further analysis from these sets of biofilm anodes can be very well linked to existing literature.

### Microbial current production and acetate uptake

The *j* of all two-chamber reactors (R1-3) increase nearly identical in the 1^st^ batch cycle and reach their first maximums at ca. 0.2 mA cm^−2^ after ca. 60 h (Fig 2). Subsequently, *j* of R1 slightly increases but *j* of R2 and R3 decrease and form 2^nd^ maximums during the 1^st^ batch cycle. Although the current density profiles are considerably different for R1-3, the slopes of decreasing acetate concentration are comparable for all three reactors (Fig 2 and S5 Fig in S1 File). Notably, acetate concentration increases in R3 from 0.2 to 1.1 mM at the end of the 1^st^ batch cycle, which is not observed for R1 and R2. The 2^nd^ batch cycles of R1-3 clearly progress more similar in terms of initial *j* increase, initial acetate decrease, *j*_max_ (≈0.4 mA cm^−2^), and the overall *j* profile. Likewise, all reactors show a little increase in acetate concentration in the beginning of the 2^nd^ batch cycle. This is probably due to the diffusion of small amounts of hydrogen from the counter electrode chamber and related hydrogenotrophic acetogenesis (S8 Equations in S1 File). At the end of the 2^nd^ batch cycle, R3 exhibit a prolonged phase of current decrease and slower acetate decrease probably due to a stronger pH decrease in R3 compared to R1 and R2 (S7 Table in S1 File).

In general, one-chamber reactors (R4-6, Fig 3) perform more similar than two-chamber reactors (R1-3) in 1^st^ and 2^nd^ batch cycle considering initial slope, current profile, and *j*_max_ (≈0.45 mA cm^−2^). Nevertheless, at the end of the 1^st^ batch cycle, all reactors show different behaviors, i.e., they differ in their current plateaus. These plateaus are accompanied by intermediate increase of acetate concentration. As Fig 3 shows, the increases differ among R4 to R6, but the higher the increase in acetate concentration, the longer is the current plateau at the end of the batch cycle. Similar to two-chamber reactors, R4 and R6 exhibit a short increase in acetate concentration in the beginning of the 2^nd^ batch cycle that is not observed for R5.

The difference between two-chamber (R1-3) and one-chamber (R4-6) reactors can be assigned to cathodically produced hydrogen. Hydrogen serves as additional substrate in R4-6 leading to higher current production, intermediate increase of acetate concentration, and the formation of the current plateau (Fig 3). Hydrogen represents an additional energy source and can be directly consumed by few *Geobacter* spp. for current production [15,16,40]. The increase in acetate concentration, especially in the 1^st^ batch cycle of R4 and R6, suggests that the hydrogen is also consumed by other microorganisms (i.e., acetogens) for producing acetate (S8 Equations in S1 File). However, as the reactor atmosphere is nitrogen saturated and as medium does not contain bicarbonate from the beginning, acetogens rely on carbon dioxide from acetate oxidation and hydrogen produced at the cathode for acetate production. Thus, acetogens do not only provide an additional acetate source for *Geobacter* spp. but also remove bicarbonate resulting in thermodynamically improved conditions for acetate degradation. However, the considerably smaller acetate increase as well as the missing current plateau in the 2^nd^ batch cycle can be assigned to a higher amount and activity of *Geobacter* biomass from the start in combination with a wash out of acetogens due to medium exchange as the biggest share of acetogens is supposed to be in the liquid [57].

### Total coulombic efficiency

The total coulombic efficiency (CE_t_) considers acetate uptake and charge production of a complete batch cycle and represents the typically published CE value. CE_t_ of two-chamber (19.6 ±8.3%) and one-chamber (91.6 ±25.6%) reactors substantially differ in the 1^st^ batch cycle but converge in the 2^nd^ batch cycle (49.3 ±13.2% for two-chamber reactors and 68.3 ±0.7% for one-chamber reactors) (Table 3). The difference can be certainly assigned to the consumption of cathodically produced hydrogen, either immediately or via acetate production, in one-chamber reactors. CE_t_ of two-chamber reactors is apparently low compared to literature values (Table 1). Typically, CE_t_ of ≥90% were reported for pure cultures of *Geobacter sulfurreducens* as well as for *Geobacter* spp. enriched biofilms (Table 1). However, most of the available literature data on CE_t_ refers to mature *Geobacter* spp. biofilms, i.e., biofilms cultivated for several days up to several weeks. As the metabolic activity of *Geobacter* spp. cells embedded in biofilms is subject to acetate diffusion, proton transport, as well as available redox potential [58,59], biofilm growth is limited. When biofilm reaches its limiting thickness due to these physical constraints, growth reactions are minimized and only a comparable small share of carbon and electrons from acetate degradation is still utilized for cellular maintenance (i.e., preserving the cellular and biofilm structure). In this quasi-steady state, for regenerating NAD^+^ used in acetate oxidation reactions, electrons are continuously transferred to the anode resulting in a high CE_t_ (Table 1). In contrast, in the present study, the CE_t_ is determined for early stage *Geobacter* spp. enrichment biofilms. Thus, the major share of electrons from acetate degradation is used for production of biomass (i.e., cells and extracellular polymeric substances) resulting in a comparable low CE_t_. Geelhoed et al. conducted one of the rare studies analyzing CE_t_ during the beginning of *Geobacter* spp. biofilm cultivation and their reported CE_t_ (see Table 1) are similar to the values derived in the present study [35]. Nevertheless, it cannot be excluded that competing processes (e.g., acetoclastic methanogenesis and alternative electron acceptors) contributed to the low CE_t_.

**Table 3:** List of total coulombic efficiencies (CE_t_) determined in this study.

	1^st^ batch cycle	2^nd^ batch cycle
One-chamber reactors	91.6 ±25.6%	68.3 ±0.7%
Two-chamber reactors	19.6 ±8.3%	49.3 ±13.2%

### Incremental coulombic efficiency

The incremental CE (CE_i_) is calculated by dividing the produced charge with the theoretical charge from acetate oxidation of the respective sampling time interval (here 6 h, see Eq 3). When acetate increases within a time interval due to acetogenesis, CE_i_ is not shown as negative values are obtained. In the start-up phase (0 h to 75 h) of two-chamber reactors (R1-3), acetate is rapidly consumed (−0.0142±0.0021 mM Ac^−^ h^−1^ cm^−2^, S5 Fig in S1 File) but CE_i_ remains low (<40%) suggesting that biomass formation is the dominating process in the beginning of the batch cycle. The CE_i_ of R1-3 suddenly increases after 75 h to 60–140% when acetate is almost completely removed (Fig 2). One might speculate that *Geobacter* spp. switch metabolic activity from biomass production (i.e., maximizing biomass yield) to current production (i.e., maximizing metabolic rates). Furthermore, the capabilities of *Geobacter* spp. to store electrons [47] and acetate [46] could also contribute to the delay between acetate uptake and current production leading to apparent high and scattering CE_i_. Time-resolved CE_t_ is also low at the beginning of the 1^st^ batch cycle and followed by an increase and a subsequent saturation (S11 Fig in S1 File). As CE_t_ is based on consecutively summed values of acetate concentration and current, it cannot reflect comparable small changes thereof like CE_i_. A putative metabolic transition during the 1^st^ batch cycle can also be illustrated with a time-resolved comparison of theoretical charge based on acetate uptake with the experimental charge production (S12 Fig in S1 File). In the 1^st^ batch cycle, the maximums of theoretical charge and produced charge exhibit a delay of 1–2 days in all two-chamber reactors. Interestingly, this delay is not observed for the 2^nd^ batch cycle (S12 Fig in S1 File) suggesting that the *Geobacter* spp. enrichment biofilms matured during the 1^st^ batch cycle. Thus, directly from the beginning of the 2^nd^ batch cycle, a greater proportion of electrons from acetate oxidation is transferred to the anode and not used for biomass production or storage processes. One may speculate that in case of long starvation times (i.e., in absence of substrate), storage processes dominate microbial activity after substrate replenishment. Therefore, a low CE_i_ would be observed in the beginning of a batch cycle that is followed by an increase of CE_i_ after recovering of microbial activity.

During both batch cycles, CE_i_ profiles of R1-3 roughly follow corresponding *j* profiles (Fig 2). The higher *j*, the higher is CE_i_ suggesting that biomass formation rate is limiting and more electrons from acetate degradation have to be transferred to the anode for maintaining a high acetate uptake rate. During acetate oxidation, NAD(P)H is produced and subsequently used for anabolic processes [60]. If anabolic reactions are slow, NAD(P)H accumulate and less NAD(P)^+^ is available for catabolic reactions [61]. Consequently, the lack of available intracellular electron carriers leads to a slowdown of acetate oxidation and in turn acetate uptake. Therefore, the direction of more electrons from NAD(P)H to EET pathways would lead to an increased regeneration rate of NAD(P)^+^ being beneficial for acetate uptake and acetate oxidation but resulting in higher CE_i_.

In the beginning of 2^nd^ batch cycle, CE_i_ of R1-3 exhibit a rapid increase that is followed by a steadily decrease to 20% in case of R1 and R2 but the CE_i_ of R3 scatters around 60% (Fig 2). This difference can probably be assigned to different pH changes during batch cultivation. Whereas pH of R1 and R2 decrease to 6.4–6.5, pH of R3 drops to pH 6.0. This stronger pH decrease is accompanied by slower acetate uptake representing a slowdown of metabolic activity and apparently a fixation of the metabolic efficiency as the CE_i_ remains at a similar range.

The CE_i_ of one-chamber reactors (R4-6) cannot be straightforwardly interpreted as cathodically produced hydrogen and acetate production by acetogens therefrom apparently influenced calculations leading to considerable scattering of data points (Fig 3). Nevertheless, in the 2^nd^ batch cycle (i.e., after wash out of acetogens), the course of CE_i_ followed the current trend similar to the behavior of two-chamber reactors (R1-3).

### Determining Michaelis-Menten parameters

**Fig 4:**
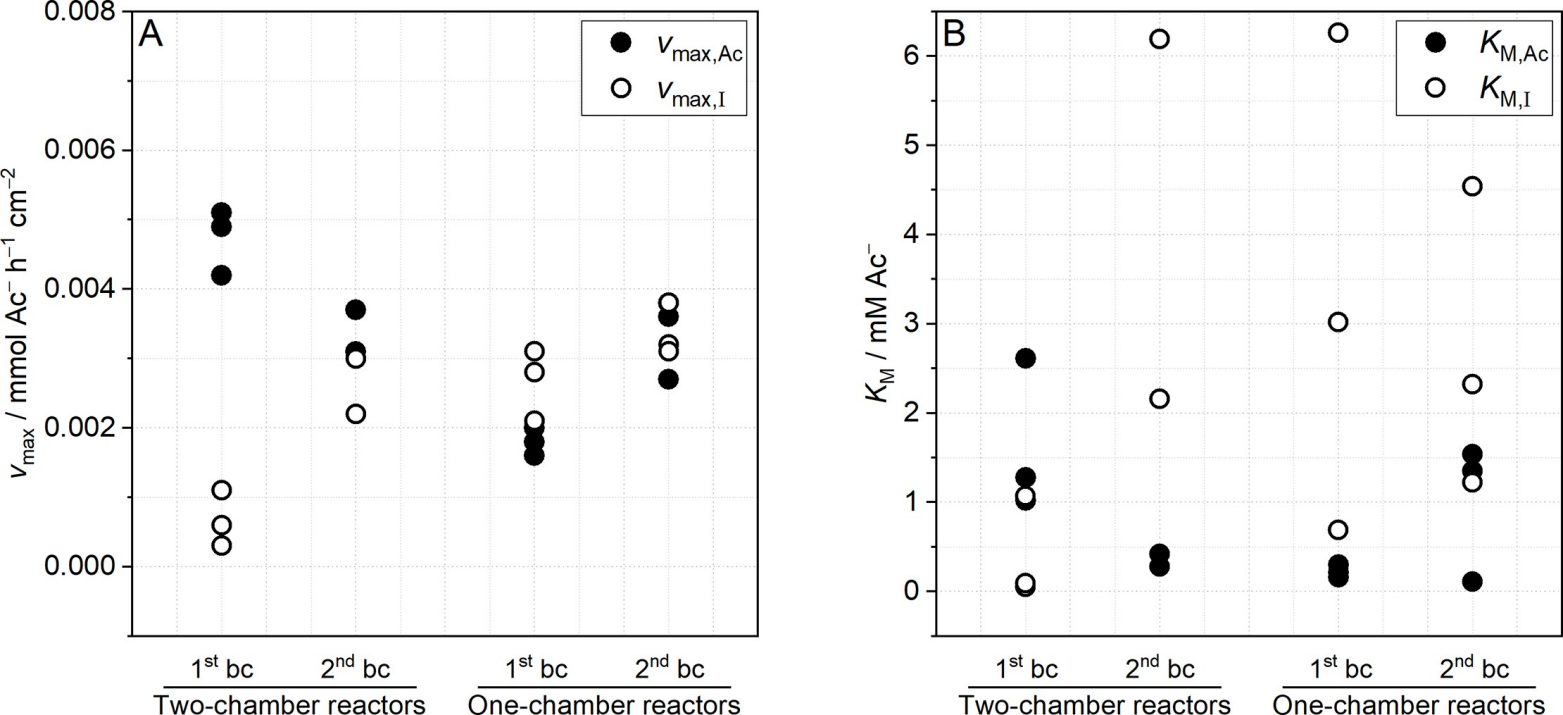
Dot plots of (A) maximum substrate uptake rate (*v*_*max*,*i*_) and (B) half-saturation concentration (*K*_*M*,*i*_) based on either acetate uptake (closed circles) or current production (open circles) derived from Michaelis-Menten kinetics regression analysis of two- and one-chamber reactors. As the prolonged current decrease and slower acetate uptake of R3 impaired application of Michaelis-Menten kinetics, respective results were omitted from this data comparison. bc: batch cycle.

Michaelis-Menten kinetics is applied for deriving the physiological parameters half-saturation concentration (*K*_M_) and maximum substrate uptake rate (*v*_max_) of *Geobacter* spp. enrichment biofilms cultivated in one- and two-chamber reactors (Fig 4). Please note that the determined *K*_M,i_ and *v*_max,i_ values represent apparent Michaelis-Menten constants being subject to substrate depletion, biofilm thickness, biofilm density, surface area of electrodes, and convection. The analysis is restricted to time periods of maximal biofilm activity, i.e., maximal acetate uptake rate (S5 and S6 Figs in S1 File). Furthermore, the acetate uptake rate and current production are normalized to amount of consumed/ transferred acetate equivalents (i.e., expressed in mmol Ac^−^ h^−1^ cm^−2^, see Eqs 4 and 5). This allows a comparison of “input” (*v*_max,Ac_, i.e., acetate uptake rate) and “output” (*v*_max,I_, i.e., current production being normalized to acetate equivalents) of acetate or electrons. In general, regression analysis with Michaelis-Menten equation yielded reasonable coefficients of determination (e.g., *R*^2^ ≥ 0.89 for *v*_max,Ac_ of two-chamber reactors) but experimental outliers were also observed (e.g., *R*^2^ = 0.14–0.75 for *v*_max,I_ during 1^st^ batch cycle of two-chamber reactors) (see S13 and S14 Figs in S1 File for regression analyses of all reactors and all batch cycles).

In case of two-chamber reactors (R1-3), the rate based on acetate uptake (*v*_max,Ac_ = 0.0042–0.0051 mmol Ac^−^ h^−1^ cm^−2^) is clearly faster than the rate derived from current production (*v*_max,I_ = 0.0003–0.0011 mmol Ac^−^ h^−1^ cm^−2^) in the 1^st^ batch cycle (Fig 4A). This difference reflects the low CE_t_ during early biofilm development (see Table 3) and indicates energy requirements for biomass production and a certain cellular capacity for acetate [46] and electron storage [47]. During the 2^nd^ batch cycle, *v*_max_ of both approaches converge (*v*_max,Ac_ ≈ *v*_max,I_ = 0.0022–0.0037 mmol Ac^−^ h^−1^ cm^−2^) suggesting maturing biofilms that shift metabolism to more current production (i.e., improving metabolic rate) and less biomass formation while keeping acetate uptake at an almost similar rate. The half-saturation concentration based on acetate uptake, *K*_M,Ac_, decreases from 1^st^ to 2^nd^ batch cycle (from 1.02–2.61 mM Ac^−^ to 0.28–0.42 mM Ac^−^) indicating an increased affinity of the maturing biofilm for acetate that results in a more effective acetate uptake. In contrast, *K*_M,I_ based on current production increases from 1^st^ to 2^nd^ batch cycle (from 0.09–1.07 mM Ac^−^ to 2.16–6.19 mM Ac^−^) (Fig 4A). In homogeneous enzyme kinetics, an increase in *K*_M_ indicates an inhibition of the reaction, which is putatively not the case here. However, the increase in *K*_M,I_ could also reflect substrate depletion in active biofilm layers as this was already demonstrated in bioelectrochemistry based on heterogeneous enzyme kinetics (e.g., immobilized enzymes at electrodes) [62]. Furthermore, the differences in *K*_M,Ac_ and *K*_M,I_ suggest that acetate transport within biofilm is affected by an increased biofilm density and thickness. Higher amounts of acetate are needed for establishing acetate saturation within the whole biofilm especially in the metabolic active layers [58,59].

In the 1^st^ batch cycle, *v*_max,Ac_ (0.0016–0.0020 mmol Ac^−^ h^−1^ cm^−2^) and *v*_max,I_ current production (0.0021–0.0031 mmol Ac^−^ h^−1^ cm^−2^) derived from one-chamber reactors (R4-6) are more comparable than for two-chamber reactors (Fig 4B). In the 2^nd^ batch cycle, *v*_max,Ac_ as well as *v*_max,I_ increase resulting in almost similar values (0.0027–0.0038 mmol Ac^−^ h^−1^ cm^−2^). *K*_M,Ac_ show a fair accordance in the 1^st^ batch cycle (0.16–0.30 mM Ac^−^) and seemingly increase in 2^nd^ batch cycle (0.11–1.54 mM Ac^−^) (Fig 4B). In contrast, for one-chamber reactors, *K*_M,I_ show a considerable variation during both batch cycles (1^st^: 0.69–6.26 mM Ac^−^, 2^nd^: 1.22–4.54 mM Ac^−^) presumably due to different mass transfer regimes and unsteady appearance of additional substrates (i.e., cathodically produced hydrogen and acetate by acetogenesis). Please note that *v*_max,Ac_, *v*_max,I_, *K*_M,Ac_ and *K*_M,I_ determined for one-chamber reactors are biased by cathodically produced hydrogen and acetogenic acetate production.

Whereas the determined *v*_max,Ac_, *v*_max,I_, and *K*_M,Ac_ values show an acceptable accordance for one- and two-chamber reactors during both batch cycles, *K*_M,I_ values considerably scatter in the majority of experiments. These variations indicate that biofilm thickness as well as biofilm density of experimental replicates progressed differently and apparently affect acetate transport (i.e., diffusion and migration), acetate storage, and electron storage. In contrast to these plausible variations in transport and storage processes, the observation of remarkably uniform *v*_max,Ac_ and *v*_max,I_ values for biofilm anodes in one- and two-chamber reactors suggest a certain robustness of processes related to acetate uptake and current production and a relative independence of these processes from acetate transport processes within the biofilm. Especially noticeable is the difference between *K*_M,Ac_ and *K*_M,I_ (Fig 4B) that is also suggesting an influence of acetate transport on theses parameters. In the present study, *K*_M,I_ is generally higher than *K*_M,Ac_ indicating that higher amounts of acetate are needed for establishing acetate saturation within the biofilm. In contrast, Lee at al. described a decrease of *K*_M_ when substrate diffusion processes were considered in their model-assisted study [44].

Nevertheless, *v*_max,Ac_, *v*_max,I_, *K*_M,Ac_ and *K*_M,I_ derived in this study from two-chamber reactors can hardly be compared to available literature data (Table 1) as i) experiments described in literature were conducted in one-chamber reactors not considering the impact of cathodically produced hydrogen, ii) Michaelis-Menten parameters are calculated on the basis of current production neglecting energy demands for biomass production and maintenance as well as the influence of acetate and electron storage, and iii) parameters that certainly influence the parameters are not standardized, e.g., biofilm age, cultivation temperature, anode potential and anode surface/reactor volume (e.g., see the effect of fiber brush anodes on *v*_max,I_ calculations in Zhu et al. [45], Table 2). These issues clearly underline the need for standardization of experimental design, conduction of experiments, and data interpretation for an accurate determination of physiological parameters of *Geobacter* spp. biofilms and other EAM [63].

## Conclusions & outlook

By combining a simple bioelectrochemical batch cultivation of *Geobacter* spp. enrichment biofilms in one- and two-chamber reactors with time-resolved substrate analysis, information on incremental coulombic efficiency (CE_i_) and Michaelis-Menten parameters are obtained. Thereby, results of one-chamber reactors are biased by cathodically produced hydrogen and intermediate acetate production. The progress of CE_i_ of early stage *Geobacter* spp. enrichment biofilms was reported for the first time. The results indicate that *Geobacter* spp. switch metabolism between optimizing metabolic rate and biomass yield depending on acetate concentration and the progress of biofilm formation. However, CE_i_ is certainly affected by acetate and electron storage processes resulting in a considerable scatter thereof. The application of Michaelis-Menten kinetics yields *v*_max,Ac_ and *K*_M,Ac_ based on acetate uptake as well as *v*_max,I_ and *K*_M,I_ based on current production. Whereas *v*_max,Ac_ and *K*_M,Ac_ were determined for the first time, few literature on *v*_max,I_ and *K*_M,I_ is available. However, comparison with available literature data is challenging as related experiments were conducted in one-chamber reactors (Table 2), and thus were biased by cathodic hydrogen formation. The obtained *K*_M,Ac_ values of biofilm anodes cultivated in two-chamber reactors seem reasonable as they are in a similar magnitude compared to *K*_M_ values of other anaerobic microorganisms like, for instance, acetoclastic methanogens (0.4–1.2 mM) [64–66], denitrifiying (0.09 mM) [67] and sulfate-reducing (0.07–0.60 mM) [64,66] bacteria (see S15 Table in S1 File for details). Focusing on *v*_max,i_, certain differences between both approaches are observed in the 1^st^ batch cycle of two-chamber reactors but values converge in 2^nd^ batch cycle. Nevertheless, more and longer cultivation experiments are necessary to analyze the progress of *v*_max,Ac_ during maturation of *Geobacter* spp. biofilms and to finally assess the influence of biomass production and maintenance as well as acetate and electron storage on the ratio of acetate uptake rate and current production. The requirement of a better data fundament is also illustrated by the observed differences between *K*_M,Ac_ and *K*_M,I_ derived from two-chamber and one-chamber reactors certainly demonstrating that transport and storage processes influence *Geobacter* spp. biofilm properties.

However, the scattering of obtained results indicate the need for a more defined experimental system and broader experimental foundation for obtaining more reliable physiological parameters of *Geobacter* spp. biofilms. This includes i) studies under steady-state conditions to minimize the influence of pH, e.g., by application of a flow cell [44], ii) inclusion of modeling approaches [68] or spatial-temporal analysis, iii) comparison of *Geobacter* spp. pure and enrichment cultures to assess the influence of minor species in the biofilm, and iv) minimally invasive measuring the biomass with, e.g., optical coherence tomography for an improved normalization of the derived parameters [69]. Especially the experimental setup (two-chamber systems), age of the examined biofilms (early stage vs. mature biofilms), and anode potential (i.e., different EET pathways) [70–72] are of utmost importance. Our results expectedly indicate that the physiology of early stage *Geobacter* spp. enrichment biofilms differ from mature biofilms. Consequently, investigations on the physiological development of electroactive biofilms should be intensified for, e.g., optimizing organic load during start-up phase of MET [48,49]. Furthermore, microbial community analysis of the bulk liquid [73] as well as headspace gas analysis will be helpful for assessing and quantifying competitive microbial processes, especially in *Geobacter* spp. enrichment biofilms.

## Supporting information

S1 File (DOCX)
