# Supplementary material for: Determining incremental coulombic efficiency and physiological parameters of early stage Geobacter spp. enrichment biofilms
Source: PLoS One. 2020 Jun 19;15(6):e0234077. doi: 10.1371/journal.pone.0234077 (PMC7304624; doi:10.1371/journal.pone.0234077)
Supplement: S1 File — (DOCX) [file pone.0234077.s001.docx]

**Determining incremental coulombic efficiency and physiological parameters of early stage *Geobacter* spp. enrichment biofilms**

**Benjamin Korth^1^, Jörg Kretzschmar^2^, Manuel Bartz^1^, Anne Kuchenbuch^1^, Falk Harnisch^1^**

**^1^Department of Environmental Microbiology, Helmholtz Centre for Environmental Research - UFZ, Leipzig, Saxony, Germany**

**^2^Biochemical Conversion Department, DBFZ Deutsches Biomasseforschungszentrum gemeinnützige GmbH, Leipzig, Saxony, Germany**

**Corresponding author: Falk Harnisch (**[falk.harnisch@ufz.de](mailto:falk.harnisch@ufz.de)**)**

## Table of content

[List of used symbols and constants 2](#_Toc30073037)

[S1 Literature data. Michaelis-Menten parameters 2](#_Toc30073038)

[S2 Figure. Michaelis-Menten analysis of data derived from Zhu et al. (2013) 4](#_Toc30073039)

[S3 Figure. Michaelis-Menten analysis of data derived from Kretzschmar et al. (2016) 5](#_Toc30073040)

[S4 Figure. Michaelis-Menten analysis of data derived from Zarabadi et al. (2019) 6](#_Toc30073041)

[S5 Figure. Linear regression analysis of acetate concentration in two-chamber reactors 6](#_Toc30073042)

[S6 Figure. Linear regression analysis of acetate concentration in one-chamber reactors 7](#_Toc30073043)

[S7 Table. pH values 8](#_Toc30073044)

[S8 Equations. Acetate oxidation by *Geobacter* spp. and acetate formation by acetogens 8](#_Toc30073045)

[S9A Figure. TRFLP analysis with HaeIII 8](#_Toc30073046)

[S9B Figure. TRFLP analysis with RsaI 9](#_Toc30073047)

[S10A Figure. Evolution of formal potential during cultivation 10](#_Toc30073048)

[S10B Figure. Exemplary cyclic voltammogram 11](#_Toc30073049)

[S11 Figure. Time-resolved theoretical and actual charge production of two-chamber reactors 12](#_Toc30073050)

[S12 Figure. Michaelis-Menten regression analysis of two-chamber reactors 13](#_Toc30073051)

[S13 Figure. Michaelis-Menten regression analysis of one-chamber reactors 14](#_Toc30073052)

[S14 Table. Literature data on Michaelis-Menten parameters of anaerobic microorganisms 15](#_Toc30073053)

[References 15](#_Toc30073054)

## List of used symbols and constants

| $A$ | Anode area, cm^2^ |
| --- | --- |
| $C_{\mathrm{Ac}}$ | Acetate concentration, mol L^−1^ |
| $\Delta C_{\mathrm{Ac}}$ | Change in acetate concentration, mol L^−1^ |
| $C_{S}$ | Substrate concentration, mol L^−1^ |
| CE | Coulombic efficiency; % |
| CE_i_ | Incremental coulombic efficiency; % |
| CE_t_ | Total coulombic efficiency (equivalent to CE), % |
| $E_{A}$ | Anode potential, V |
| $F$ | Faraday constant, 96485.3 C mol^−1^ |
| $I$ | Current, A |
| $j$ | Current density, mA cm^−2^ |
| $j_{\max}$ | Maximum current density, mA cm^−2^ |
| $K_{M}$ | Half-saturation concentration, mol L^−1^ |
| $K_{M,Ac}$ | Half-saturation concentration based on acetate uptake rate, mol L^−1^ |
| $K_{M,I}$ | Half-saturation concentration based on current production normalized to uptake rate of acetate equivalents, mol L^−1^ |
| ${\Delta n}_{S}$  $q$ | Amount of consumed substrate, mol  Charge, C |
| $t$ | Time, s |
| $V$ | Volume, L |
| $v$ | Substrate uptake rate, s^−1^ |
| $v_{\max}$ | Maximum substrate uptake rate, s^−1^ |
| $v_{\mathrm{Ac}}$ | Acetate uptake rate, mmol Ac^−^ h^−1^ cm^−2^ |
| $v_{max, Ac}$ | Maximum acetate uptake rate, mmol Ac^−^ h^−1^ cm^−2^ |
| $v_{I}$ | Output rate of acetate equivalents based on current production, mmol Ac^−^ h^−1^ cm^−2^ |
| $v_{max, I}$ | Maximum output rate of acetate equivalents based on current production, mmol Ac^−^ h^−1^ cm^−2^ |
| $z$ | Number of transferred electrons per molecule |

## S1 Literature data. Michaelis-Menten parameters

Lee et al. derived $K_{M}$ = 119-184 gCOD m^−3^ and $v_{\max}X_{f}$ = 936-1120 kgCOD m^−3^ d^−1^ by conducting experiments in a one-chamber flow reactor ($V$ = 0.26 L) and analyzing the results with the Nernst-Monod model (see Table 1 in [1]). $X_{f}$ is biomass density (50000 gVS m^−3^). The anode consisted of two graphite rods ($L$ = 4.7 cm, $d$ = 0.4 cm). By assuming that the complete rods served as anodes, an anode area of 12.3 cm^2^ is calculated.

Further assumptions:

- 1 gCOD corresponds to 1 gO_2_
- The molecular mass of oxygen is 32 g mol^−1^
- 1 molecule O_2_ accepts 4 electrons
- Oxidation of 1 molecule acetate (Ac^−^) yields 8 electrons

$K_{M}$ and $v_{\max}$ are converted via equations S1 and S2, respectively. One value is exemplified, the others are likewise calculated.

| $K_{M}=\frac{119 \frac{\mathrm{gCOD}}{m^{3}}}{32 \frac{\mathrm{gCOD}}{\mathrm{mol}}\times2}=1.86 \mathrm{mM}\mathrm{Ac}^{-}$ | (S $1$) |
| --- | --- |

| $v_{max,I}=\frac{936000 \frac{\mathrm{gCOD}}{m^{3} d}\times0.00026 m^{3}}{32 \frac{\mathrm{gCOD}}{\mathrm{mol}}\times2\times24 \frac{h}{d}\times12.3 \mathrm{cm}^{2}}=12.88 \mathrm{mmol}\mathrm{Ac}^{-}\mathrm{cm}^{-2} h^{-1}$ | (S $2$) |
| --- | --- |

Zhu et al. performed repeated batch cultivation with a fiber brush anode ($A$ = 0.22 m^2^ total fiber surface area) in a one-chamber reactor ($V$ = 28 mL) [2]. The acetate concentration and current data was extracted from Figure 6 of their article via Plot Digitizer (<http://plotdigitizer.sourceforge.net>) and current was converted via equation S 3. Subsequently, the data was fitted with Michaelis-Menten kinetics (see equation 1 in the main manuscript) (S2 Fig).

| $v_{max,I}=\frac{I\times3600\frac{s}{h}}{F\times A\times z}$ | (S $3$) |
| --- | --- |

Kretzschmar et al. cultivated a *Geobacter* spp. dominated biofilm anode in a flow cell ($V$ = 0.1 L) [3] and current was converted via equation S 4. Subsequently, the data was fitted with Michaelis-Menten kinetics (S3 Fig).

| $v_{max,I}=\frac{j\times3600\frac{s}{h}}{F\times z}$ | (S $4$) |
| --- | --- |

Zarabadi et al. cultivated *Geobacter sulfurreducens* PCA biofilm anode in a one-chamber reactor ($V$ = 0.1 L) [4] and current was converted via equation S 4. Subsequently, the data was fitted with Michaelis-Menten kinetics (S4 Fig).

## S2 Figure. Michaelis-Menten analysis of data derived from Zhu et al. (2013)





S2 Fig Michaelis-Menten analysis of data derived from Figure 6 of Zhu et al. (2013).

## S3 Figure. Michaelis-Menten analysis of data derived from Kretzschmar et al. (2016)


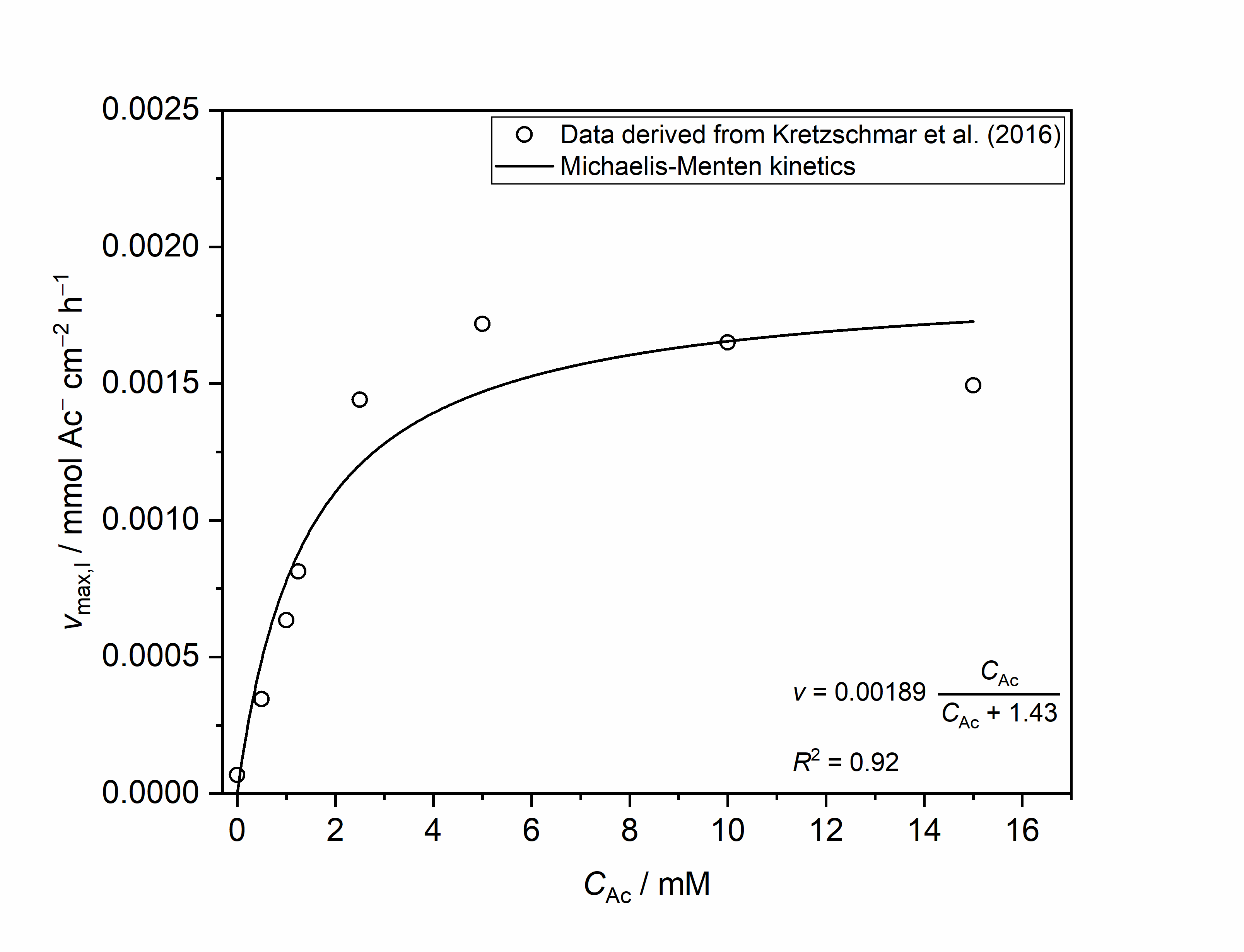


S3 Fig Michaelis-Menten analysis of data derived from Figure 3 of Kretzschmar et al. (2016).

## S4 Figure. Michaelis-Menten analysis of data derived from Zarabadi et al. (2019)


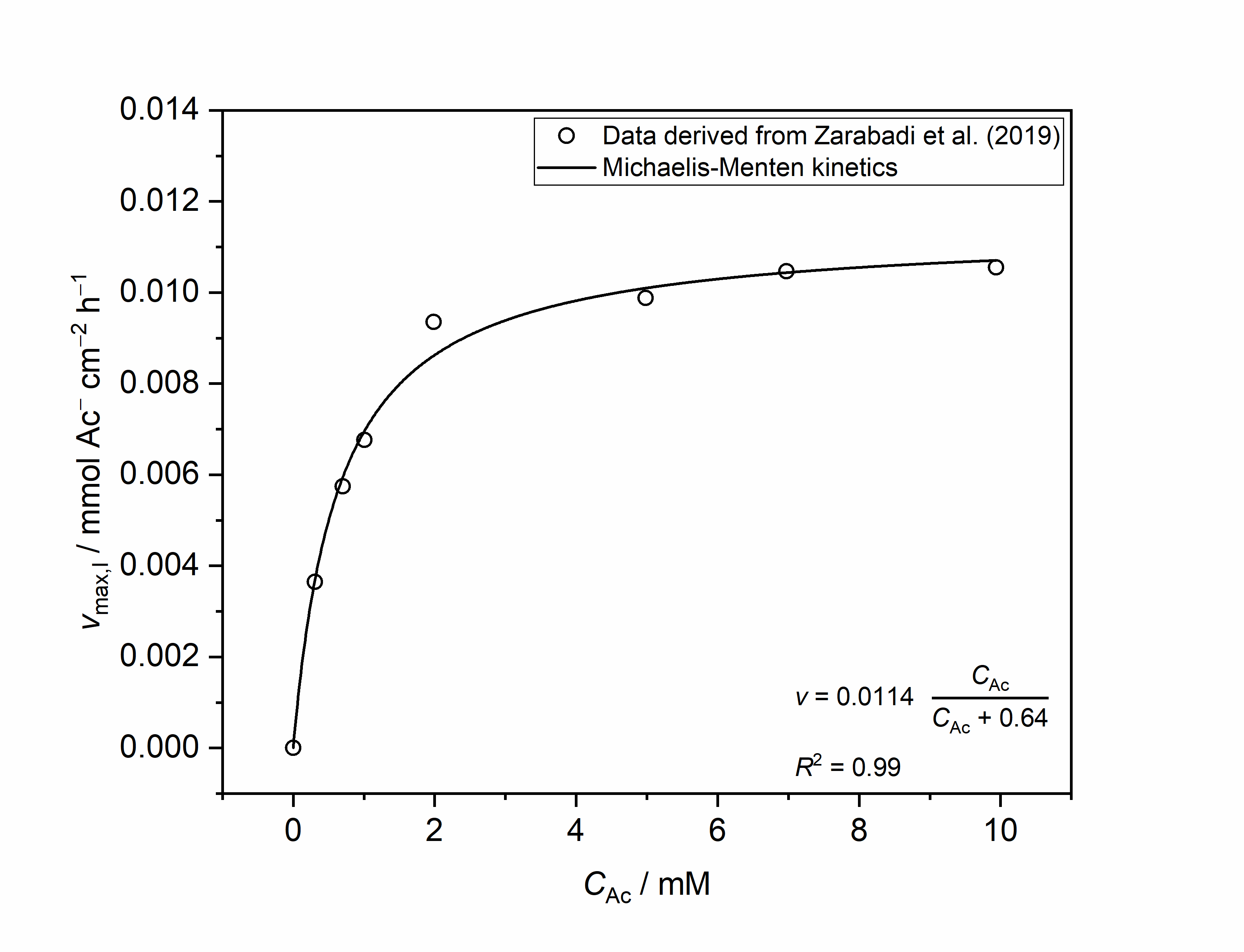


S4 Fig Michaelis-Menten analysis of data derived from Figure S5b of Zarabadi et al. (2019).

## S5 Figure. Linear regression analysis of acetate concentration in two-chamber reactors

By performing linear regression analysis of acetate concentration, time periods of maximal biofilm activity, i.e., maximal acetate uptake rate, were identified. When linear regression was *R*^2^ ≥ 0.99, maximal acetate uptake rate was assumed for data points used for the linear regression and for data points following linear regression. The same definition for maximal acetate uptake rate was used for Figure S6.


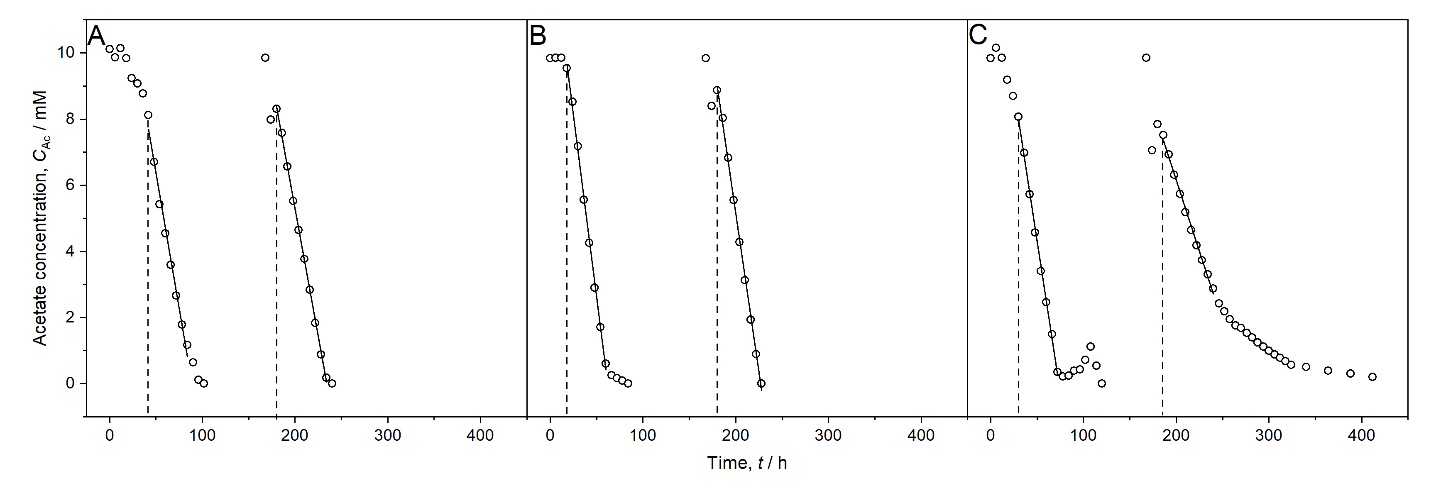


S5 Fig Course of acetate concentration in two-chamber reactors during 1^st^ and 2^nd^ batch cycle (bc). Open circles: experimental data, solid lines: linear regression of constant acetate decrease. (A) R1. 1^st^ bc: y = −0.1637x + 14.5592, R^2^ = 0.988. 2^nd^ bc: y = −0.1543x + 36.1534, R^2^ = 0.999 (B) R2. 1^st^ bc: y = −0.2198x + 13.6020, R^2^ = 0.998. 2^nd^ bc: y = −0.1919x + 43.5417, R^2^ = 0.998 (C) R3. 1^st^ bc: y = −0.1834x + 13.4844, R^2^ = 0.998. 2^nd^ bc: y = −0.0862x + 23.4061, R^2^ = 0.995. Vertical dashed lines indicate the first data point that was used for Michaelis-Menten analysis.

## S6 Figure. Linear regression analysis of acetate concentration in one-chamber reactors


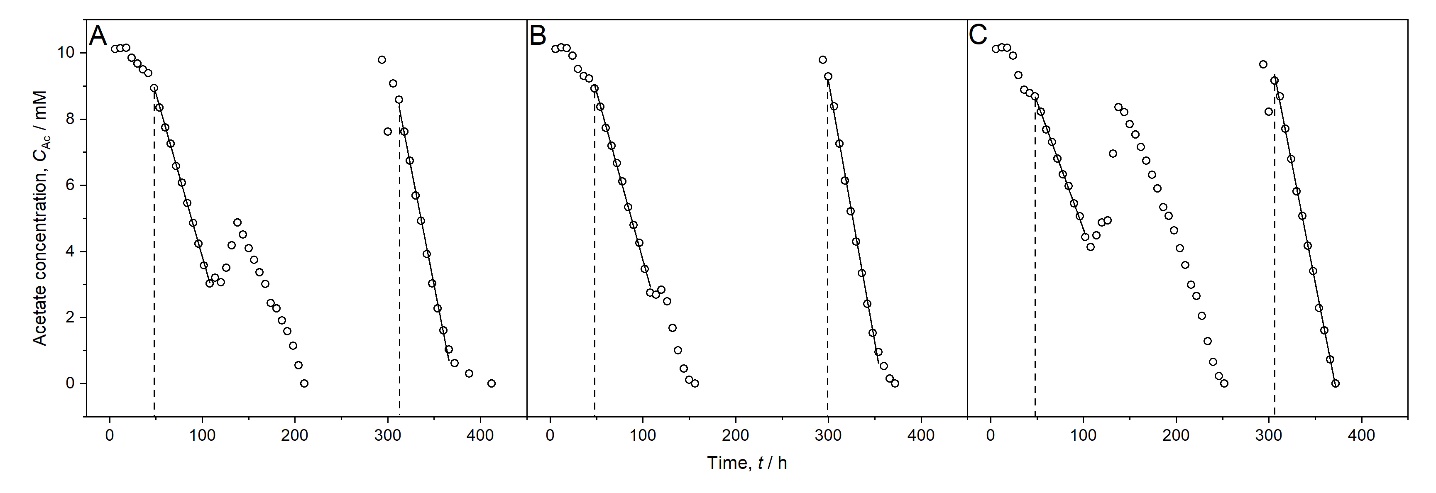


S6 Fig Course of acetate concentration in one-chamber reactors during 1^st^ and 2^nd^ batch cycle (bc). Open circles: experimental data, solid lines: linear regression of constant acetate decrease. (A) R4. 1^st^ bc: y = −0.0986x + 13.6943, R^2^ = 0.999. 2^nd^ bc: y = −0.1428x + 52.9516, R^2^ = 0.995 (B) R5. 1^st^ bc: y = −0.1016x + 13.8895, R^2^ = 0.998. 2^nd^ bc: y = −0.1581x + 56.5653, R^2^ = 0.997 (C) R6. 1^st^ bc: y = −0.0768x + 12.3540, R^2^ = 0.999. 2^nd^ bc: y = −0.1432x + 53.1721, R^2^ = 0.999. Vertical dashed lines indicate the first data point that was used for Michaelis-Menten analysis.

## S7 Table. pH values

| **Reactor** | **1^st^ batch cycle** | | **2^nd^ batch cycle** | |
| --- | --- | --- | --- | --- |
|  | **Initial pH** | **Final pH** | **Initial pH** | **Final pH** |
| **R1** | 7.0 | 6.7 | 7.0 | 6.4 |
| **R2** | 7.0 | 6.8 | 7.0 | 6.4 |
| **R3** | 7.0 | 6.6 | 7.0 | 6.0 |
| **R4** | 7.0 | 7.1 | 7.0 | 7.1 |
| **R5** | 7.0 | 7.1 | 7.0 | 7.1 |
| **R6** | 7.0 | 7.0 | 7.0 | 7.2 |

## S8 Equations. Acetate oxidation by *Geobacter* spp. and acetate formation by acetogens

Acetate oxidation: $\mathrm{CH}_{3}\mathrm{COO}^{-}+{4H}_{2}O\to{2HCO}_{3}^{-}+{9H}^{+}+{8e}^{-}$

Acetate formation: ${2HCO}_{3}^{-}+4H_{2}+H^{+}\to\mathrm{CH}_{3}\mathrm{COO}^{-}+{4H}_{2}O$

## S9A Figure. TRFLP analysis with *Hae*III


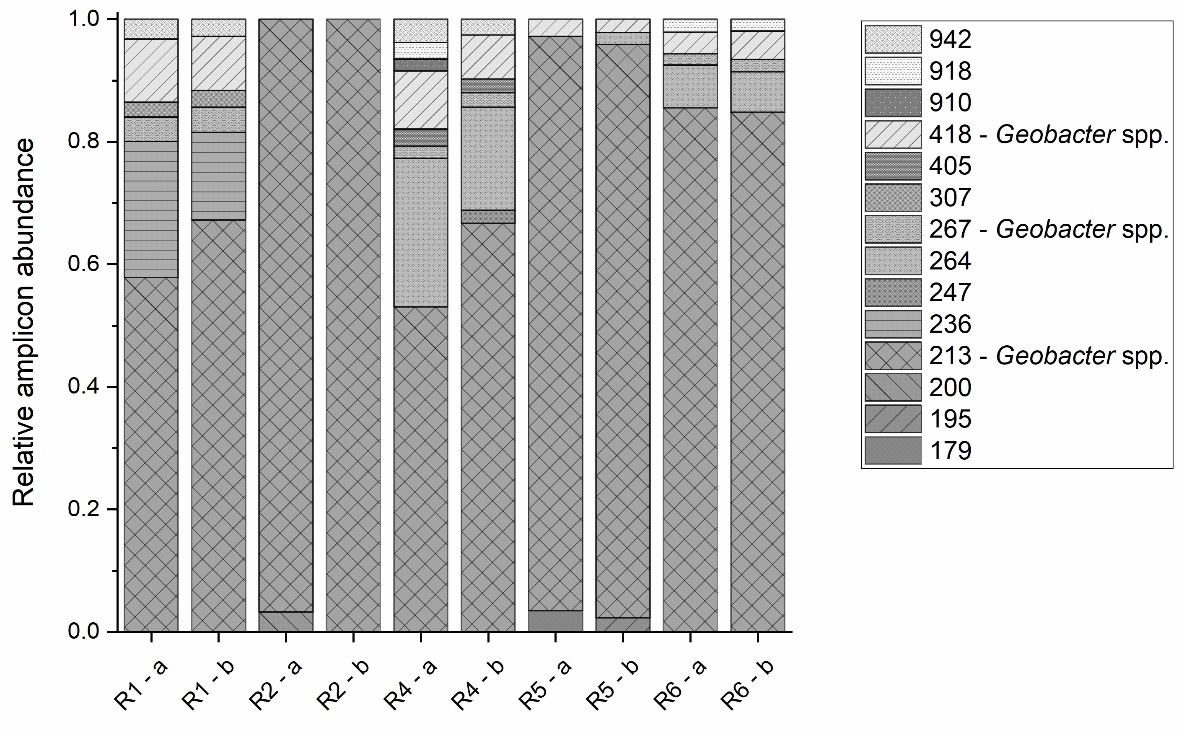


S9A Fig TRFLP analysis of biofilm anodes from two-chamber (R1-2) and one-chamber (R4-6) reactors. a and b represent technical replicates, i.e., from every reactor, two biofilm samples from different anode areas were taken. PCR Products were digested with HaeIII. The legend comprises all detected fragments (in base pairs, bp) and the fragments with 213, 267, and 418 bp represent Geobacter spp. (see S9B Fig). R3 could not be sampled as the biofilm completely detached from the anode due to an electrical short at the end of cultivation.

According to Koch et al., *Geobacter anodireducens* SD-1 is the present *Geobacter* species in the used *Geobacter* spp. enrichment biofilms [5]. The available sequence (CP014963.1) indicates that in addition to the terminal restriction fragment (TRF) 213 bp, also TRF 267 bp and 418 bp belong to *Geobacter anodireducens* SD-1 and probably occur due to incomplete enzymatic digestion as the sequence contains the enzymatic cleavage sites (CS, green) at the respective positions (grey: primers for PCR reaction, see SI-1.8):

GAGTTTGATCCTGGTTCAGAACGAACGCTGGCGGCGTGCCTAACACATGC UniBac27f

AAGTCGAACGTGATCCGCCCTTCGGGGTGGTGAAAGTGGCGCACGGGTGA 100

GTAACGCGTGGATAATCTGCCCGAGGATTTGGGATAACATCTCGAAAGGG

GTGCTAATACCGAATAAGCCCACGGGGTCTACGGATCTTGCGGGAAAAGG 200

GGGGGACCTTCGGGCCTCCTGTCCTTGGATGAGTCTGCGTACCATTAGCT CS 214 bp

AGTTGGTGGGGTAATGGCCTACCAAGGCGACGATGGTTAGCTGGTCTGAG CS 267 bp 300

AGGATGATCAGCCACACTGGAACTGAGACACGGTCCAGACTCCTACGGGA

GGCAGCAGTGGGGAATTTTGCGCAATGGGGGAAACCCTGACGCAGCAACG 400

CCGCGTGGGTGATGAAGGCCTTCGGGTCGTAAAGCTCTGTCTGGAGGGAA CS 418 bp

GAAATGATTGGGAGCTAATACCTCTTGATCTTGACGGTACCTCTGAAGGA 500

AGCACCGGCTAACTCCGTGCCAGCAGCCGCGGTAATACGGAGGGTGCAAG

CGTTGTTCGGAATTATTGGGCGTAAAGCGCGTGTAGGCGGTCTTTTAAGT 600

CTGATGTGAAAGCCCCGGGCTCAACCTGGGAAGTGCATTGGAAACTGGGA

GGCTTGAGTACGGGAGAGGAGAGTGGAATTCCTAGTGTAGGAGTGAAATC 700

CGTAGATATTAGGAGGAACACCGGTGGCGAAGGCGGCTCTCTGGACCGAT

ACTGACGCTGAGACGCGAAAGCGTGGGTAGCAAACAGGATTAGATACCCT 800

GGTAGTCCACGCCGTAAACGATGAGTACTAGGTGTTGCGGGTATTGACCC

CTGCAGTGCCGTAGCTAACGCATTAAGTACTCCGCCTGGGAAGTACGGTC 900

GCAAGACTAAAACTCAAAGGAATTGACGGGGGCCCGCACAAGCGGTGGAG CS 932 bp

CATGTGGTTTAATTCGACGCAACGCGCAGAACCTTACCTGGGCTTGACAT 1000

CCACGGAACCCTCCCGAAACGGAGGGGTGCCCTTCGGGGAGCCGTGAGAC

AGGTGCTGCATGGCTGTCGTCAGCTCGTGTCGTGAGATGTTGGGTTAAGT 1100

CCCGCAACGAGCGCAACCCTTGTCATCAGTTGCCATCATTCAGTTGGGCA

CTCTGATGAAACTGCCGGTGTCAAACCGGAGGAAGGTGGGGATGACGTCA 1200

AGTCCTCATGGCCCTTATGTCCAGGGCTACACACGTGCTACAATGGCCGG CS 1211 & 1246 bp

TACAAAGAGTAGCAATACCGCGAGGTGGAGCCAATCTCAGAAAGCCGGTC 1300

TCAGTTCGGATTGGAGTCTGCAACTCGACTCCATGAAGTCGGAATCGCTA

GTAATCGCGGATCAGCATGCCGCGGTGAATACGTTCCCGGGCCTTGTACA CS 1391 bp 1400

CACCGCCCGTCACACCACGGGAGTCGACTGGTCCCGAAGTGCGTGAGCTA

ACCCCTTGTGGGAGGCAGCGTCCTAAGGAATGGTCGGTGACTGGGGTGAA Univ1492r 1500

GTCGTAACAAGGTAGCCGTA

## S9B Figure. TRFLP analysis with *Rsa*I


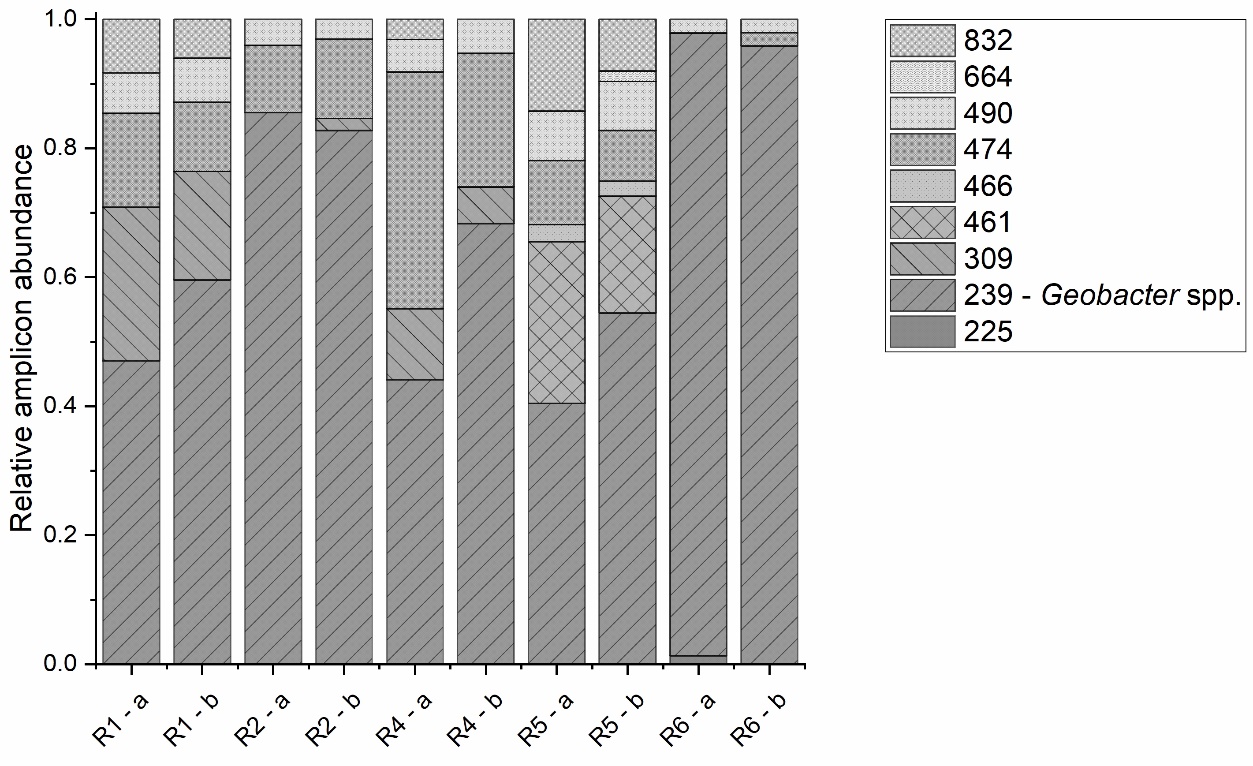


S9B Fig TRFLP analysis of biofilm anodes from two-chamber (R1-2) and one-chamber (R4-6) reactors. PCR Products were digested with RsaI. The legend comprises all detected fragments (in base pairs, bp). Similar to the signal pattern derived from the digestion with HaeIII (S8A Fig), one major fragment (239 bp) is present representing Geobacter spp. as it was demonstrated by Harnisch et al. [6]. a and b represent technical replicates, i.e., from every reactor, two biofilm samples from different anode areas were taken. R3 could not be sampled as the biofilm completely detached from the anode due to an electrical short at the end of cultivation.

## S10A Figure. Evolution of formal potential during cultivation


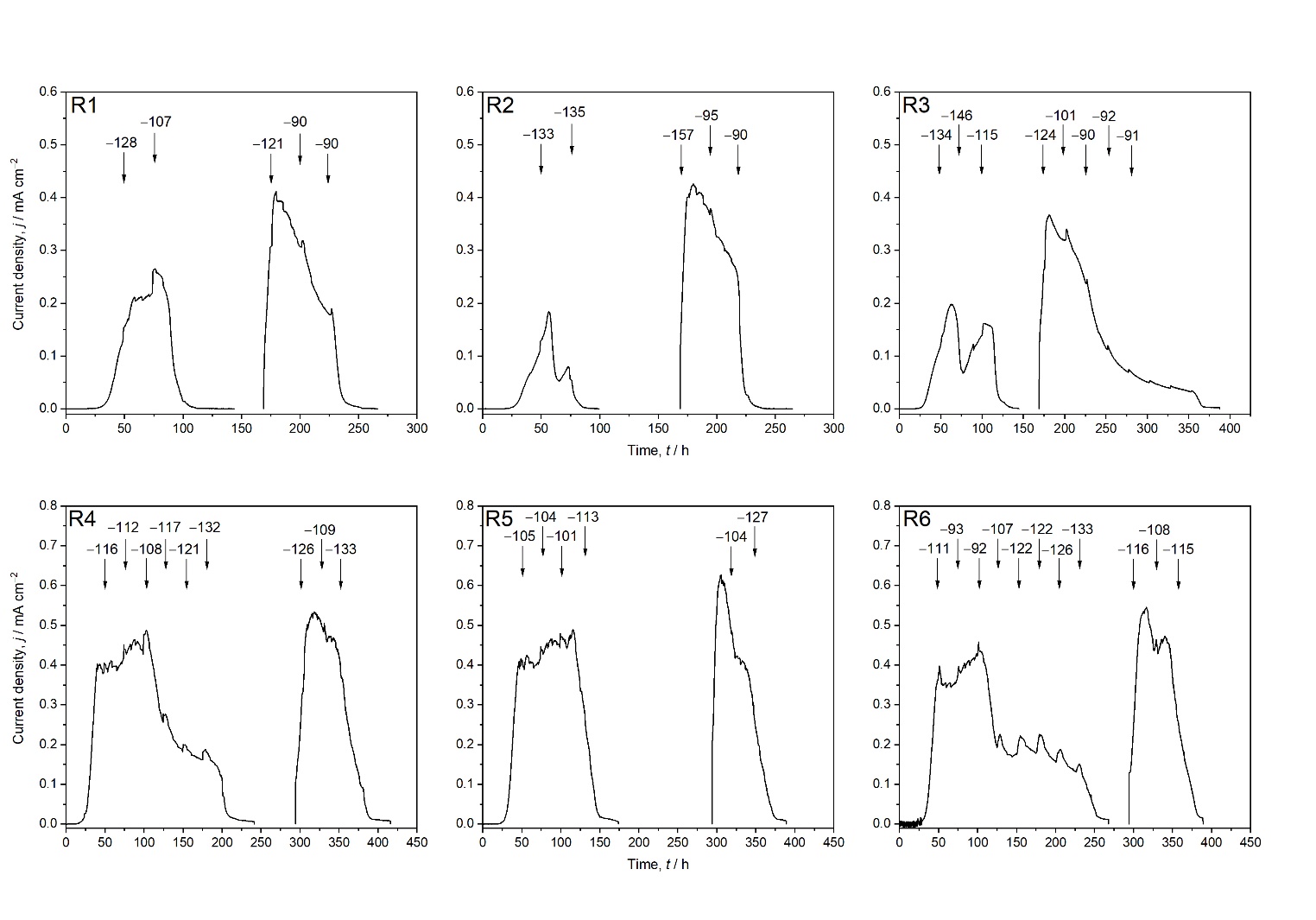


S10A Fig Progress of current density ($j$) and formal potentials determined by cyclic voltammetry (CV) of two-chamber (R1-3) and one-chamber (R4-6) reactors. Arrows indicate execution of CV and the determined formal potential in mV (vs. SHE). For analysis, the 1^st^ derivative of the third CV scan was formed (see S9B Fig). The data was smoothened (moving average, n = 10), and the mean of maximums from forward and backward scan were calculated. The analysis was performed with OriginPro 2018G SR1 Version b9.5.1.195.

The difference in formal potential between the beginning and the end of a batch cycle in two-chamber reactors can be partially explained with pH variation during a batch cycle and the redox-Bohr effect [7]. It can be considered by applying the Nernst equation (equation S 5). This is exemplified for the 2^nd^ batch cycle of R1 (equation S 6).

| $E_{\mathrm{comp}}=E+\frac{RT}{zF}\ln\frac{c_{\mathrm{ox}}}{c_{\mathrm{red}}}$ | (S $5$) |
| --- | --- |

- $E_{\mathrm{comp}}$: formal potential compensated for pH
- $E$: determined formal potential at the end of the 2^nd^ batch cycle (S10A Fig R1)
- $R$: universal gas constant (8.314 J mol^−1^ K^−1^)
- $T$: temperature (308.15 K)
- $z$: number of transferred electrons ($z$ = 1)
- $F$: Faraday constant (96485.3 C mol^−1^)
- $c_{\mathrm{ox}}$: concentration of oxidized form (derived from pH at the beginning of the 2^nd^ batch cycle,S6 table)
- $c_{\mathrm{red}}$: concentration of reduced form (derived from pH at the end of the 2^nd^ batch cycle, S6 table)

| $E_{\mathrm{comp}}=-90 \mathrm{mV}+\frac{8.314 \frac{J}{mol K}\times308.15 K}{96485.3 \frac{C}{\mathrm{mol}}}ln\frac{{10}^{-7}}{{10}^{-6.4}}$  $=-90 \mathrm{mV}+27 \mathrm{mV}\times\left( -1.38 \right)=-127.3 \mathrm{mV}$ | (S $6$) |
| --- | --- |

$E_{\mathrm{comp}}$ is in good agreement with the formal potential determined at the beginning of the 2^nd^ batch cycle of R1 (121 mV).

## S10B Figure. Exemplary cyclic voltammogram


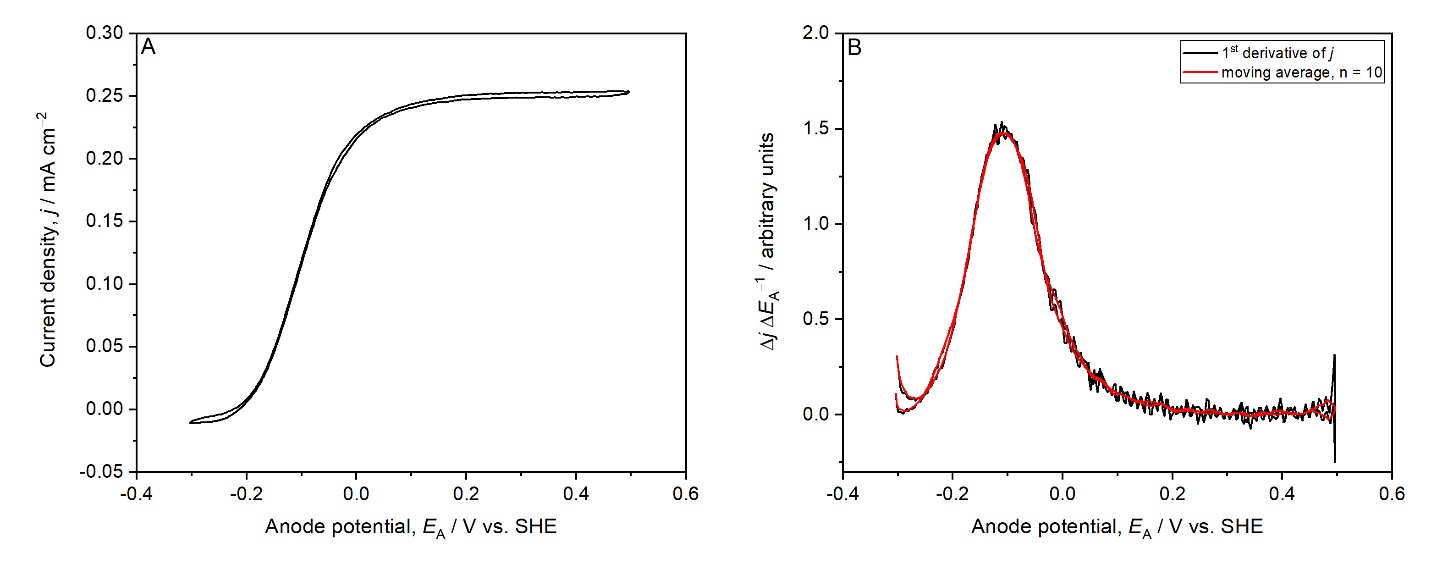


S10B Fig (A) Cyclic voltammetry data from R1 recorded after ca. 76 h. The third scan is depicted. (B) 1^st^ derivative of the third scan (black line). Data was smoothened (red line, adjacent-averaging, points of window = 10) in order to determine the mean of maximums of forward and backward scan. The analysis was performed with OriginPro 2018G SR1 Version b9.5.1.195.

## S11 Figure. Time resolved total coulombic efficiency (CE_t_)


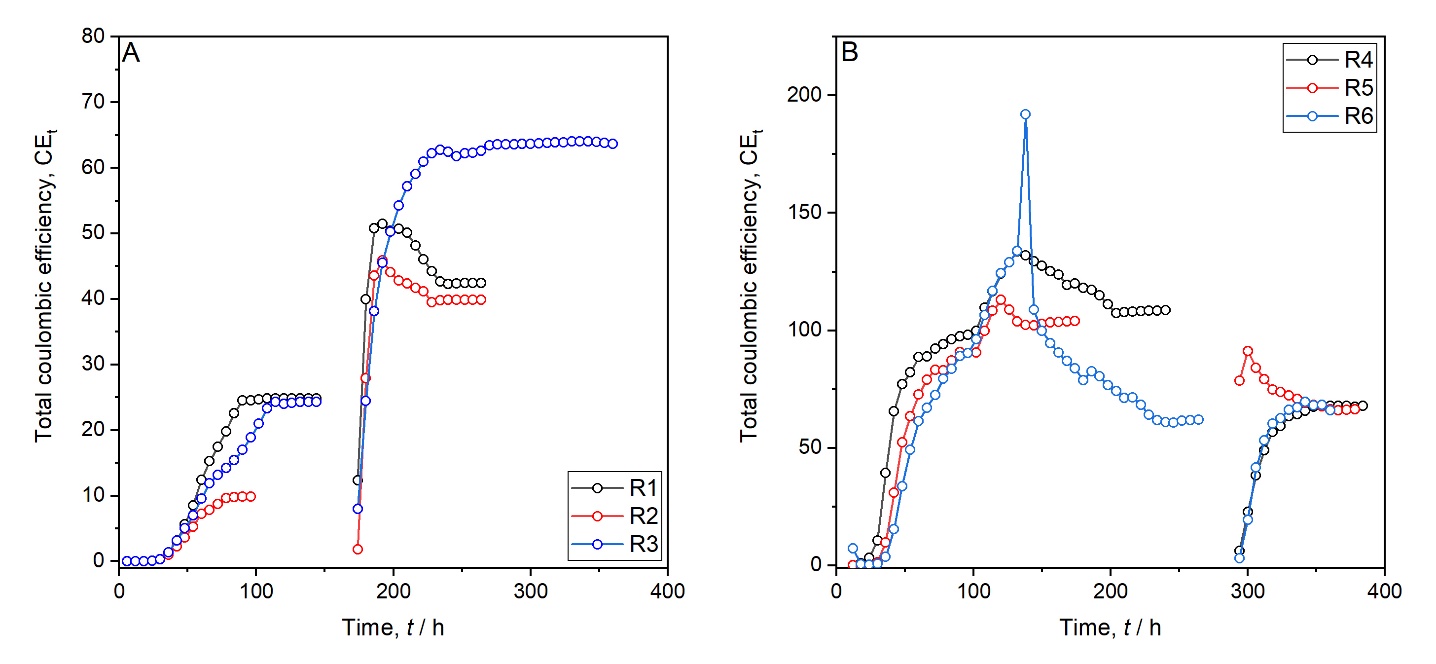


S11 Fig Time-resolved total coulombic efficiency (CE_t_) of (A) two-chamber reactors and (B) one-

chamber reactors. Open circles indicate sampling events.

## S12 Figure. Time-resolved theoretical and actual charge production of two-chamber reactors


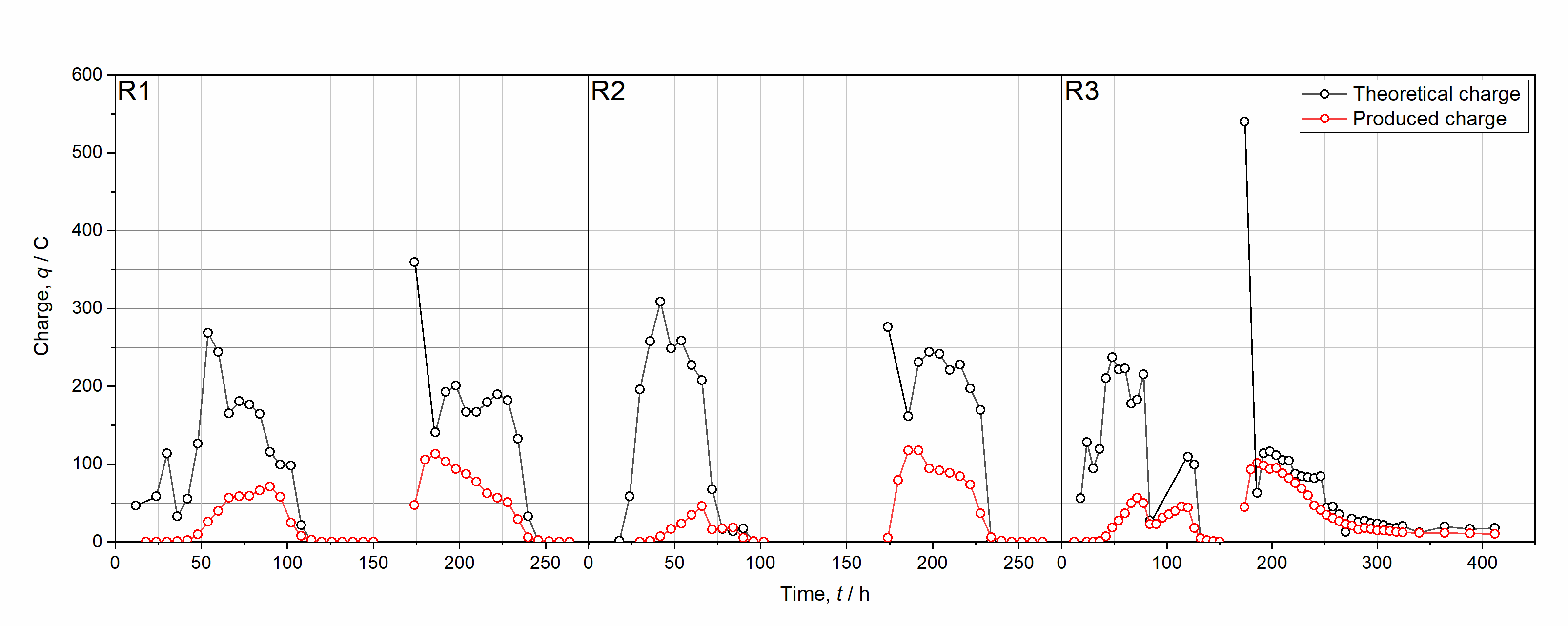


S12 Fig Time course of theoretical and actual charge production of two-chamber reactors during 1^st^ and 2^nd^ batch cycle. Dark solid line: Theoretical charge production based on acetate uptake and oxidation. Red solid line: Derived charge production from current integration. Open circles indicate sampling events.

## S13 Figure. Michaelis-Menten regression analysis of two-chamber reactors


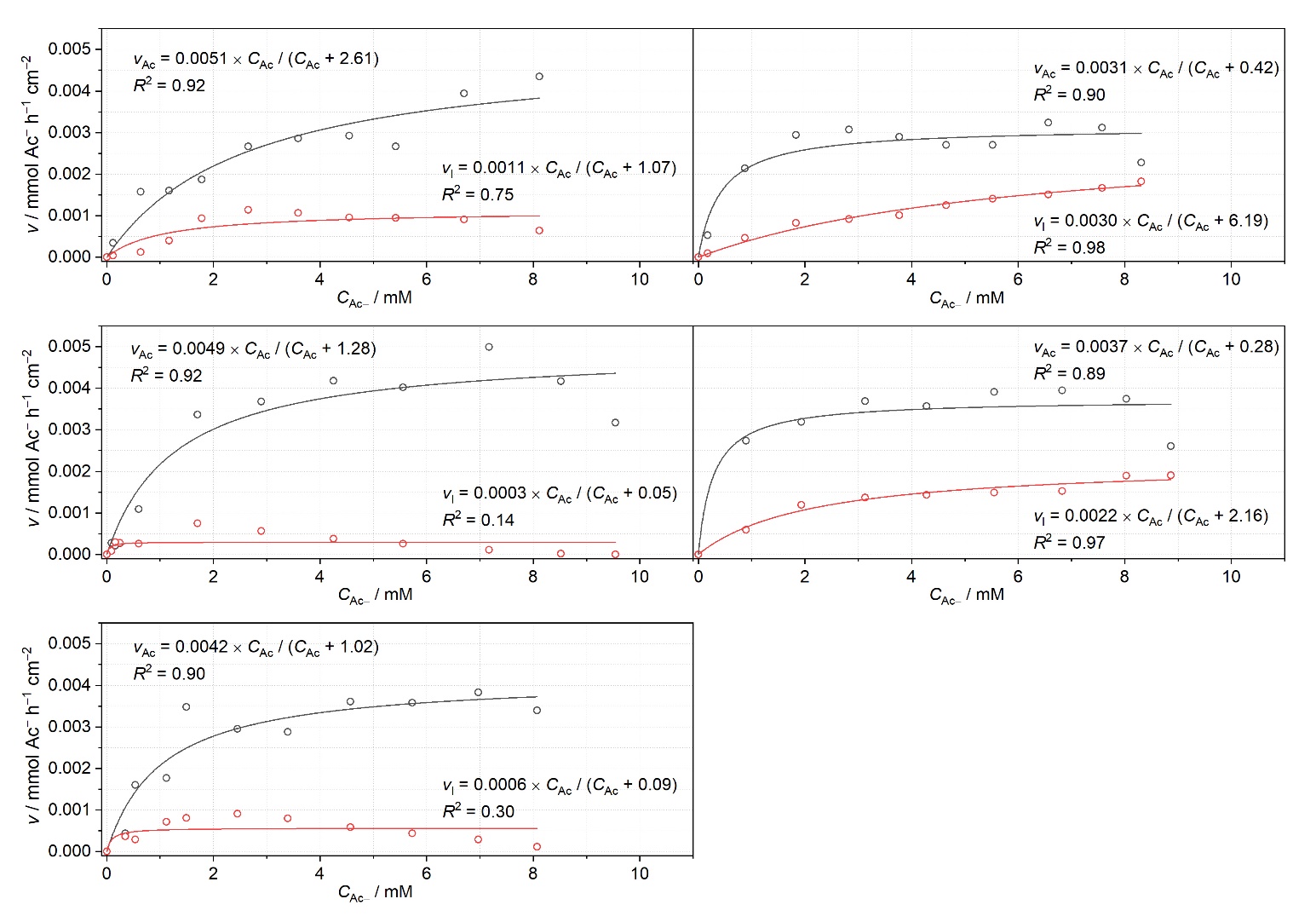


S13 Fig Michaelis-Menten regression analysis of two-chamber reactors.$v_{Ac}$: acetate uptake rate, $v_{I}$: C. (A) R1, 1^st^ batch cycle. (B) R1, 2^nd^ batch cycle. (C) R2, 1^st^ batch cycle. (D) R2, 2^nd^ batch cycle. (E) R3, 1^st^ batch cycle. 2^nd^ batch cycle of R3 was not analysed as the prolongend current decrease and slower acetate consumption impaired application of Michaelis-Menten kinetics. Black open circles: experimental data of $v_{Ac}$, black solid lines: regression analysis of $v_{Ac}$. Red open circles: experimental data of $v_{I}$, red solid lines: regression analysis of $v_{I}$. Regression analysis was performed with OriginPro 2018G SR1 Version b9.5.1.195.

## S14 Figure. Michaelis-Menten regression analysis of one-chamber reactors


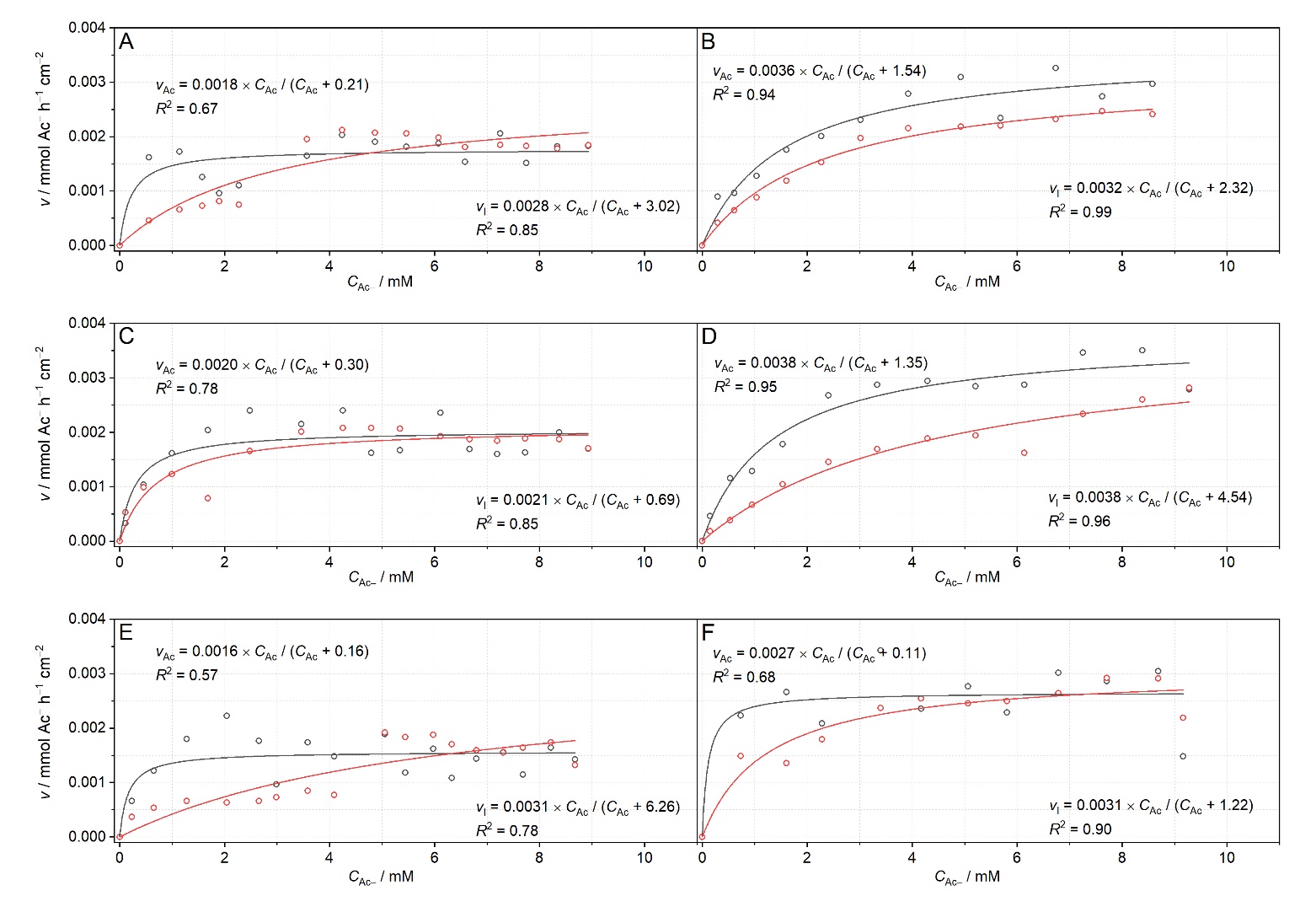


S14 Fig Michaelis-Menten regression analysis of one-chamber reactors.$v_{Ac}$: acetate uptake rate, $v_{I}$: uptake rate of acetate equivalents based on current production. (A) R4, 1^st^ batch cycle. (B) R4, 2^nd^ batch cycle. (C) R5, 1^st^ batch cycle. (D) R5, 2^nd^ batch cycle. (E) R6, 1^st^ batch cycle. (F) R6, 2^nd^ batch cycle. Black open circles: experimental data of $v_{Ac}$, black solid lines: regression analysis of $v_{Ac}$. Red open circles: experimental data of $v_{I}$, red solid lines: regression analysis of $v_{I}$. Regression analysis was performed with OriginPro 2018G SR1 Version b9.5.1.195.

## S15 Table. Literature data on Michaelis-Menten parameters of anaerobic microorganisms

| **Process** | **Reaction** | ***K*_M_** | ***v*_max_** | **Reference** |
| --- | --- | --- | --- | --- |
| **Nitrate reduction** | Enrichment culture | 0.09 mM | 1.2×10^−4^ mM s^−1^ | [8] |
| **Sulfate reduction** | Enrichment culture | 0.51 mM | 5.7×10^−4^ mM s^−1^ | [9] |
|  | *Desulfobacter postgatei* | 0.07-0.023 mM | 53 µmol min^−1^ g^−1^ | [10] |
|  | *Desulfobacca acetoxidans* | 0.6±0.4 mM | 43±14 µmol min^−1^ g^−1^ | [11] |
|  | *Desulforhabdus amnigenus* | 0.6±0.4 mM | 28±7 µmol min^−1^ g^−1^ | [11] |
| **Acetoclastic Methanogenesis** | Enrichment culture | 0.46 mM | 9.5×10^−4^ mM s^−1^ | [9] |
|  | *Methanosaeta soehngenii* | 0.4-0.7 mM | 76 µmol min^−1^ g^−1^ | [11] |
|  | *Methanosaeta concilii* | 0.8-1.2 mM | 32 µmol min^−1^ g^−1^ | [11] |

## References

1. Lee H-S, Torres CI, Rittmann BE. Effects of Substrate Diffusion and Anode Potential on Kinetic Parameters for Anode-Respiring Bacteria. Environ Sci Technol. 2009;43(19):7571–7. doi: 10.1021/es9015519.

2. Zhu X, Tokash JC, Hong Y, Logan BE. Controlling the occurrence of power overshoot by adapting microbial fuel cells to high anode potentials. Bioelectrochemistry. 2013;90:30–5. doi: 10.1016/j.bioelechem.2012.10.004.

3. Kretzschmar J, Rosa LFM, Zosel J, Mertig M, Liebetrau J, Harnisch F. A microbial biosensor platform for in-line quantification of acetate in anaerobic digestion: potential and challenges. Chem Eng Technol. 2016;39(4):637–42. doi: 10.1002/ceat.201500406.

4. Zarabadi MP, Couture M, Charette SJ, Greener J. A generalized kinetic framework for whole‐cell bioelectrocatalysis in flow reactors clarifies performance enhancements. ChemElectroChem. 2019;6(10):2715–8. doi: 10.1002/celc.201900732.

5. Koch C, Popiel D, Harnisch F. Functional Redundancy of Microbial Anodes fed by Domestic Wastewater. ChemElectroChem. 2014;1(11):1923–31. doi: 10.1002/celc.201402216.

6. Harnisch F, Koch C, Patil SA, Hübschmann T, Müller S, Schröder U. Revealing the electrochemically driven selection in natural community derived microbial biofilms using flow-cytometry. Energy Environ Sci. 2011;4(4):1265–7. doi: 10.1039/C0EE00605J.

7. Morgado L, Paixão VB, Schiffer M, Pokkuluri PR, Bruix M, Salgueiro CA. Revealing the structural origin of the redox-Bohr effect: the first solution structure of a cytochrome from Geobacter sulfurreducens. Biochem J. 2012;441(1):179–87. doi: 10.1042/BJ20111103.

8. Leffelaar PA, Wessel WW. Denitrifikation in a homegeneous closed system: experiment and simulation. Soil Sci. 1988;145(5):335–49.

9. Scholten JCM, Van Bodegom PM, Vogelaar J, Van Ittersum A, Hordijk K, Roelofsen W, et al. Effect of sulfate and nitrate on acetate conversion by anaerobic microorganisms in a freshwater sediment. FEMS Microbiol Ecol. 2002;42(3):375–85. doi: 10.1016/S0168-6496(02)00359-8.

10. Schönheit P, Kristjansson JK, Thauer RK. Kinetic mechanism for the ability of sulfate reducers to outcompete methanogens for acetate. Arch Microbiol. 1982;132:285–8.

11. Oude Elferink SJWH, Luppens SBI, Marcelis CLM, Stams AJM. Kinetics of acetate oxidation by two sulfate reducers isolated from anaerobic granular sludge. Appl Environ Microbiol. 1998;64(6):2301–3.
